# Assessment of intra and interregional genetic variation in the Eastern Red-backed Salamander, *Plethodon cinereus*, via analysis of novel microsatellite markers

**DOI:** 10.1371/journal.pone.0186866

**Published:** 2017-10-20

**Authors:** Alexander C. Cameron, Jeffry J. Anderson, Robert B. Page

**Affiliations:** 1 Department of Biology, John Carroll University, University Heights, Ohio, United States of America; 2 Department of Biology, St. John’s University, Collegeville, Minnesota, United States of America; 3 Department of Science & Mathematics, Texas A&M University—San Antonio, San Antonio, Texas, United States of America; Australian National University, AUSTRALIA

## Abstract

The red-backed salamander (*Plethodon cinereus*) has long-served as a model system in ecology, evolution, and behavior, and studies surveying molecular variation in this species have become increasingly common over the past decade. However, difficulties are commonly encountered when extending microsatellite markers to populations that are unstudied from a genetic perspective due to high levels of genetic differentiation across this species’ range. To ameliorate this issue, we used 454 pyrosequencing to identify hundreds of microsatellite loci. We then screened 40 of our top candidate loci in populations in Virginia, Pennsylvania, and Ohio—including an isolated island population ~ 4.5 km off the shore of Lake Erie (South Bass Island). We identified 25 loci that are polymorphic in a well-studied region of Virginia and 11 of these loci were polymorphic in populations located in the genetically unstudied regions of Ohio and Pennsylvania. Use of these loci to examine patterns of variation within populations revealed that South Bass Island has low diversity in comparison to other sites. However, neither South Bass Island nor isolated populations around Cleveland are inbred. Assessment of variation between populations revealed three well defined genetic clusters corresponding to Virginia, mainland Ohio/Pennsylvania, and South Bass Island. Comparisons of our results to those of others working in various parts of the range are consistent with the idea that differentiation is lower in regions that were once glaciated. However, these comparisons also suggest that well differentiated isolated populations in the formerly glaciated portion of the range are not uncommon. This work provides novel genetic resources that will facilitate population genetic studies in a part of the red-backed salamander’s range that has not previously been studied in this manner. Moreover, this work refines our understanding of how neutral variation is distributed in this ecologically important organism.

## Introduction

**Fig 1:**
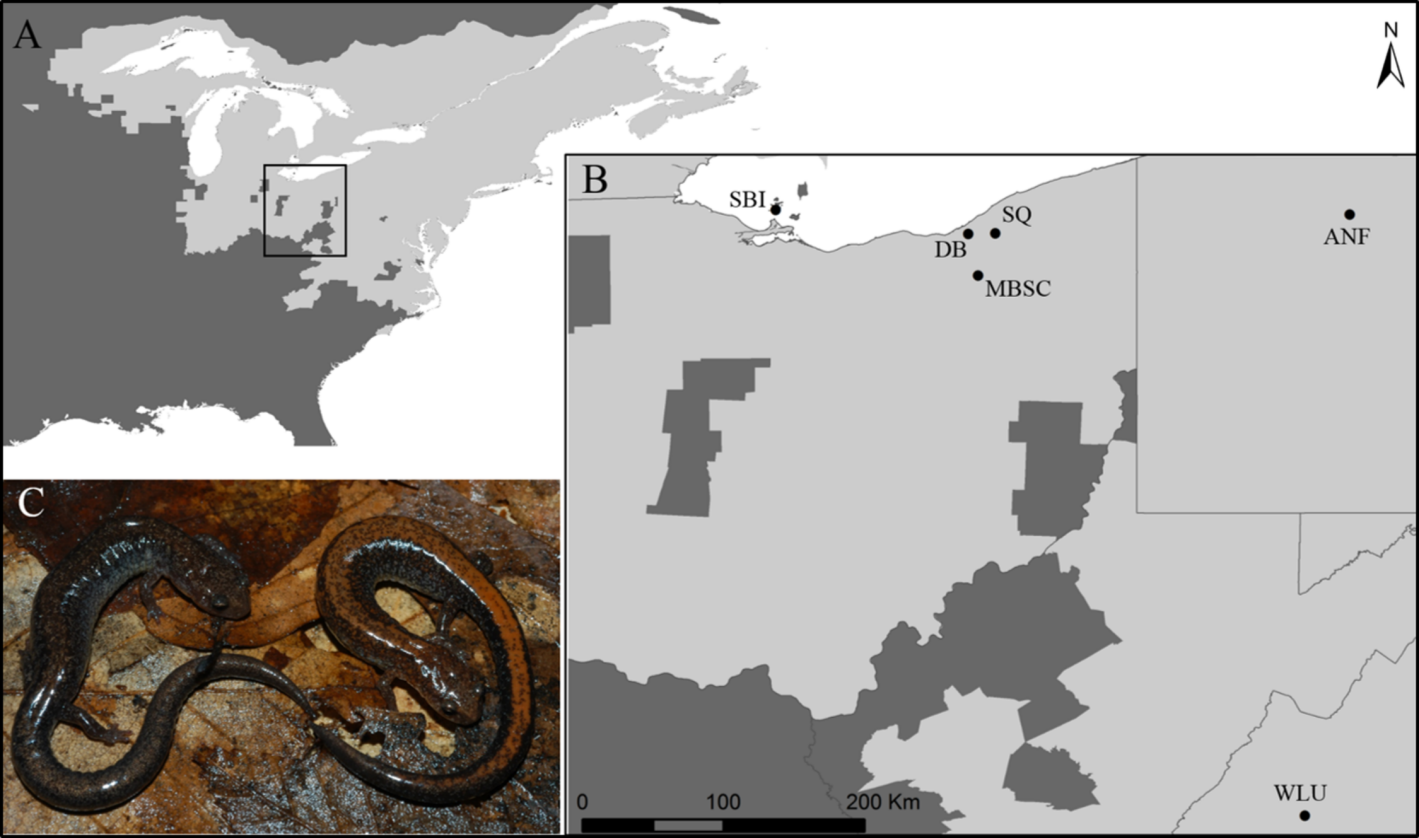
Range map, sampling localities, and color morphs of the Eastern Red-backed Salamander. (A) The geographic range (light-grey) of *Plethodon cinereus* and the focal region of the current study (black rectangle) (B) Location of the sampled localities: Washington and Lee University (WLU; VA), Allegheny National Forest (ANF; PA), Squire Valleevue and Valley Ridge Farm (SQ; OH), Manotoc Boy Scout Camp (MBSC; OH) Doan Brook (DB; OH), and South Bass Island (SBI; OH). (C) The color morphs of *P*. *cinereus* found within the sampled populations, the unstriped morph (left) and the striped morph (right).

Since the latter half of the 20^th^ century, the Eastern Red-backed Salamander (*Plethodon cinereus*; from here out ‘red-backed salamander’) has been the subject of hundreds of ecological, evolutionary, and behavioral studies [1,2]. The red-backed salamander is a fully terrestrial, direct developing species and is one of the most abundant vertebrates in eastern North America (Fig 1; [3,4]). In addition to being highly abundant, red-backed salamanders act as a top-down regulator within the detrital food web [5–7]. These attributes exemplify why the red-backed salamander has served as an excellent model for examining a wide variety of interesting topics including: territoriality [8,9], the dynamics of complex social systems [10,11], phenotypic variation [12,13], fine-scale population differentiation [14,15], and the effects of anthropogenic modifications of the landscape on gene flow [16,17].

At present, most of what is known about the molecular ecology of red-backed salamanders is based on populations in western Virginia [14–16,18–20]. However, a handful of studies have been conducted in other areas including: Quebec [17,21], Indiana [22], New York [13], and Maryland [23]. The only published study we know of investigating genetic structure across a wide swath of the red-backed salamander’s range was conducted in the 1970’s and is based on protein electrophoresis rates [24]. In this study, Highton and Webster [24] concluded that populations within Appalachian glacial refugia were markedly differentiated, even across short geographic distances, whereas populations in formally glaciated portions of the range were by-and-large genetically uniform. While synthesis across studies based on modern molecular techniques [14,17,21,22] is generally consistent with the findings of Highton and Webster ([24]; see discussion), most of the red-backed salamander’s range has not been investigated via contemporary approaches to population genetics.

Recently, several studies examining the evolutionary ecology of red-backed salamander populations in Ohio have been conducted [25–27]. However, to the best of our knowledge, nothing is known about population genetic dynamics within this part of the range and consequently no codominant markers have been validated in or identified from these populations. Although roughly 20 microsatellite loci have been isolated from *P*. *cinereus* [13,14,20], the typical number of loci used in published studies (mean number of loci = 6.33; [13–19,21–23]) is well below the number of loci used in comparable studies on other taxa [28]. The most likely explanation for this is that the red-backed salamander’s large geographic range (Fig 1) and limited capacity for dispersal [14,29] have resulted in genetic differentiation, which can lead to differing patterns of monomorphism and/or problems with PCR-based amplification when extending loci to new populations (see results in [13,16,17,23]). Consequently, there is a need for additional genetic resources for red-backed salamanders that will enable molecular ecology research in and across divergent parts of the range. To this end, we used 454 pyrosequencing to generate a genomic shotgun sequence library and informatically mined this library to identify microsatellite markers. We then used these new markers to genotype individuals from several populations in geographic regions of interest (*e*.*g*., a well-studied region in western Virginia and a region around Cleveland, Ohio that is becoming increasingly well studied from an ecological perspective) that have not been surveyed for microsatellite diversity previously.

## Materials and methods

### Study sites and tissue collection

During November 2011 Salamander tail clippings were collected from the Washington and Lee University (WLU) campus in Rockbridge County, Virginia, USA (Table 1) to facilitate 454 pyrosequencing. Between April and October 2014, additional tail clippings were collected from WLU and the other locales listed in Table 1 to facilitate marker development and PCR-based genotyping. Tail tissue was obtained by searching under cover objects such as rocks and logs. When salamanders were discovered, tail autotomy was induced by clasping the tail with forceps approximately 1 cm from the tip. Tail tips were then placed in 90%-100% molecular biology grade ethanol and animals were released at their point of capture. Samples were then stored at -20°C until DNA isolations were performed. Tissue collected in Virginia was collected under Virginia DGIF permit #33510 and Virginia DGIF permit #49225. Tissue collected in Ohio was collected under Ohio DNR permit #17–09 and tissue collected in Pennsylvania was collected under Pennsylvania Fish and Boat Commission permit #2015-01-0040. The tissue collection procedure was approved by the John Carroll University IACUC (protocol #1302).

**Table 1:** Locality and basic information for *P*. *cinereus* samples.

Site Name	ID	County	State	Longitude	Latitude	Morph^a^	N
Squire Valleevue	SQ	Cuyahoga	OH	41.498894	81.421992	S	10
Doan Brook	DB	Cuyahoga	OH	41.49383	81.593892	S	12
South Bass Island	SBI	Ottawa	OH	41.648528	82.823322	US	12
Manatoc Boy Scout Camp	MSBC	Summit	OH	41.229173	81.53055	US/S	24
Allegheny National Forest	ANF	Forest	PA	41.619184	79.159359	S	12
Washington and Lee University	WLU	Rockbridge	VA	37.794173	79.444600	S	32

Potentially amplifiable loci were identified from the Washington and Lee University population and subsequently screened for amplification reliability and genetic diversity across locales in Ohio and Pennsylvania. Manatoc Boy Scout Camp was the only population that exhibited color polymorphism and equal numbers of each morph were collected at this site.

^a^S = Striped US = Unstriped

### 454 sequencing and microsatellite identification

DNA for sequencing was isolated according to the phenol/chloroform extraction method described by Taggart *et al*. [30] and isolates were qualified and quantified via agarose gel electrophoresis and UV spectrophotometry. A single high-quality isolate was submitted to the University of Georgia Genomics Facility, where this isolate was pooled with DNA from two other species (see [31,32]) that were differentiated by terminal barcodes [33]. A library of single stranded template DNA fragments was then produced using the GS FLX titanium general library preparation kit (Roche). Initial sequencing employed the 454 GS FLX titanium sequencing kit XLR70 (Roche) run on ¼ 70 x 75 mm picotiter plate and additional sequencing employed the 454 GS FLX titanium sequencing kit XL+ (Roche) run on ½ 70 x 75 mm picotiter plate.

To identify 454 fragments that contained potentially amplifiable loci (PALs), we used MSATCOMMANDER 1.0.8 [34] to scan for repeat containing fragments. When running these searches, we required that dinucleotide and trinucleotide motifs contain ≥ eight repeat units and that tetra-hexanucleotdie motifs contain ≥ six repeat units. The default settings for MSATCOMMANDER’s PRIMER3 [35] interface were used to batch design primers, with the exceptions that G/C content was restricted to between 40 and 60% and the minimum primer melting temperature was set to 57°C.

### Microsatellite screening and genotyping

Upon identifying microsatellite containing 454 fragments, we prioritized tandem (*i*.*e*., we excluded compound and interrupted repeats) tri-pentanucleotide repeat motifs because they are frequently easier to score than dinucleotide repeats [28]. Once we arrived at a list of candidate PALs, we used the forward and reverse primer sequences of each candidate primer pair as queries in BLASTn searches of our 454 library to ameliorate the potential for redundancy. PALs whose forward and reverse primer sequences were identical to 454 fragments other than those they were designed from were precluded from molecular investigation. These searches were carried out and visualized in GENEIOUS Version R9 (Biomatters).

DNA for PCR was isolated from tail clippings using the Blood and Tissue DNeasy kit (Qiagen) according to the manufacturer’s instructions. This procedure included an RNAse digestion and resulting isolates were quantified and qualified via UV spectrophotometry and agarose gel electrophoresis. The 40 PALs selected for screening (S1 Table and S1 File) were initially investigated in the WLU population since the sample that we performed 454 sequencing on was obtained from this population. Loci that performed well in WLU were prioritized in South Bass Island (SBI) and mainland Ohio and Pennsylvania (OH/PA; DB, MBSC, SQ, ANF; see Fig 1B); however, eventually most loci were tested using SBI and OH/PA samples. In general, PCRs followed the protocol described in Schluke [36] and used the M13F(-21) sequence as a tag to facilitate 6-FAM labeling (see [31,32] for additional details). Briefly, all PCRs were 25 μl in volume and contained 1x buffer, 20 ng of template DNA, 1.5 mM MgCl_2_, 0.2 mM of each dNTP, 0.8 μM of non-M13(-21)-tagged primer, 0.8 μM of 6-FAM labeled M13(-21) primer, 0.2 μM of M13(-21)-tagged primer, and 0.625 units of GoTaq polymerase (Promega). Reaction conditions were: 94°C for 2 minutes followed by 25 cycles of (1) 94°C for 30 seconds, (2) 62°C for 30 seconds decreasing by 0.3°C per cycle and (3) 72°C for 40 seconds. These conditions were followed by 8 additional cycles of (1) 94°C for 30 seconds, (2) 53°C for 30 seconds, and (3) 72°C for 40 seconds and a final cleanup step of 72°C for 30 minutes. Genotyping reactions were run in 96 well plates that contained four duplicate reactions per locus in WLU, and on average ~ five duplicate reactions per locus per SBI and OH/PA locale. Reaction success was determined by 2% agarose gel electrophoresis and successful reactions were shipped to the Arizona State University DNA Lab where they were subjected to fragment analysis in an ABI 3730 (Life Technologies) using GENESCAN LIZ 600 as an internal sizing standard. Standard curve fitting, manual scoring, and binning were performed using GENEIOUS, Version R9 (Biomatters).

### Statistical analyses of microsatellite data

#### Summary statistics and quality control

GENALEX, Version 6.5 [37] was used to calculate a variety of summary statistics on a population-by-population basis, including: number of alleles per locus, effective number of alleles, observed heterozygosity (H_o_), and Hardy-Weinberg expected heterozygosity (H_e_). Given the uneven sample sizes among populations we additionally calculated allelic richness (A_R_) using POPGENKIT, where sample size was determined for each locus based on the smallest number of individuals sampled across all populations [38]. GENEPOP, Version 4.5.1 [39] was used to test each locus for departure from Hardy-Weinberg proportions in each population and to test each pair of loci for departure from genotypic equilibrium in each population. In addition, we used GENEPOP to compute the Weir and Cockerham [40] estimator of F_IS_ for each locus in each population. MICROCHECKER, Version 2.2.3 [41] was used to check each locus in each population for evidence of null alleles, scoring errors, and large allele drop out.

#### Bottleneck tests and effective population size estimation

We used the polymorphic loci from each locale to test for evidence of recent reductions in effective population size via the heterozygosity excess approach implemented in BOTTLENECK [42]. This approach compares H_e_ with the level of heterozygosity expected at drift-mutation equilibrium (H_eq_)—a quantity that is more sensitive than H_e_ to the loss of genetic richness that occurs during population reductions. Deviations were assessed under the stepwise mutation model (SMM), infinite alleles model (IAM), and two-phase mutation model (TPM). Following the recommendations of Piry et al. [42], under the TPM we assumed 95% of all mutations were single-step mutations with 12% of the variance within multistep mutations. We determined if there were significant deviations between H_e_ and H_eq_ using the Wilcoxon signed-rank test implemented in BOTTLENECK. In addition, the output from GENALEX was used to calculate mean *M*-ratios across polymorphic loci, which were assessed against the critical value of 0.68 recommended by Garza and Williams [43]. Effective population size (N_e_) estimates were generated using polymorphic loci for each respective locale via the linkage disequilibrium [44] and the heterozygote-excess methods [45] implemented in NeESTIMATOR v2.0.1 [46].

#### Population differentiation

We examined population differentiation using several approaches based upon a variety of conceptual and computational frameworks. First, we used GENALEX to calculate global and pairwise estimates of G_ST_ and Hedrick’s further standardized G"_ST_ [47] that were averaged across all loci. To examine large-scale patterns of differentiation, we used STRUCTURE, Version 2.34 [48] to infer the optimal value of K using the correlated allele frequencies model and allowing for admixture. We assessed K = 1–7, preforming ten replicate runs for each value of K. Each Markov Chain Monte Carlo (MCMC) simulation consisted of 500,000 iterations discarded as burn-in, with an additional 500,000 sampling iterations. The optimal value of K was determined by using STRUCTURE HARVESTER [49] to compute ΔK [50]. Replicate runs were aligned and visualized using CLUMPP [51] and DISTRUCT [52]. We then used the genetic clusters obtained from STRUCTURE to perform an AMOVA [53] in GENALEX that partitioned genetic variation among clusters, among individuals within clusters, and within individuals.

Because the algorithm implemented in STRUCTURE tends to recover the highest level of subdivision among hierarchically differentiated populations (see STRUCTURE software manual), we performed two additional analyses in STRUCTURE on the mainland OH/PA sites. When conducting these analyses, we examined K = 1–5 when considering SQ, DB, MBSC, and ANF and K = 1–4 when considering SQ, DB, and MBSC. Both of these analyses were based on the same model, number of replicate runs, and MCMC parameters detailed above for the large-scale STRUCTURE analysis.

STRUCTURE generates clusters by maximizing conformity to Hardy-Weinberg equilibrium and tends to assign individuals to superficial clusters when sampling is conducted across an isolation-by-distance (IBD) cline [54,55]. Given the degree of geographic separation among the populations we sampled and the low dispersal ability of red-backed salamanders [14], it is nearly certain that IBD is contributing to differentiation among populations (see below). Therefore, we conducted a discriminant analysis of principal components (DAPC; [56]) using the *adegenet* package [57] for the R statistical computing environment, as this approach allows for inference of patterns of differentiation without assuming Hardy-Weinberg equilibrium or a particular migration model (*i*.*e*., island, stepping stone, etc.). When performing DAPC, we used K-means clustering and the Bayesian information criterion (BIC) to assess the fit of K-means clustering solutions for K = 1–40. The cluster memberships determined via K-means were then used as prior group assignments when performing DAPC. Prior to performing DAPC we assessed the optimal number of principal components to retain using the cross-validation procedure described in Jombart and Collins [58].

Finally, we examined the relationship between genetic differentiation and geographic distance among mainland OH/PA locales in order to gain insight into the degree to which IBD influences substructure within the OH/PA cluster identified by our large-scale STRUCTURE analysis (see below). To do this, we used the GENEPOP [39] package for R to compute approximate bootstrap 95% confidence intervals for the slope of the regression of pairwise F_ST_/(1 –F_ST_) against the natural logarithm of geographic distance [59,60]. Because these samples (DB, SQ, MBSC, and ANF) were collected across a spatial scale that cannot be fairly described as “local” (see [60]), we make no effort to use the resulting slope to estimate Dσ^2^. Rather, we use the slope of this regression and the associated approximate bootstrap confidence interval to get a rough idea of the degree of correlation between genetic differentiation and geographic distance across a scale that spans ~ 10–200 km.

#### Detection of outlier loci

To examine whether there is evidence for some of the markers we discovered residing in genomic regions that have been targets of selection, we tested for outlier loci by using BAYESCAN 2.1 to implement the regression-based Bayesian framework described in Foll and Gaggiotti [61]. This approach decomposes F_ST_ into a population-specific component (β) common to all loci and a locus-specific component (α) common to all populations. This in turn enables comparisons between models containing α and β terms for a given locus (selection model) and models in which the α term for a given locus has been removed (neutral model). When running BAYESCAN we used 20 pilot runs with a length of 5000 steps, a burn in period of 100,000 steps, a sample size (number of sampled steps) of 100,000 with a thinning interval of 10, and prior odds for the neutral model of 10. This analysis was performed across all locales (Fig 1), and the q-values provided in the BAYESCAN output were used to achieve an FDR of 0.05.

## Results

### 454 sequencing and PAL discovery

**Fig 2:**
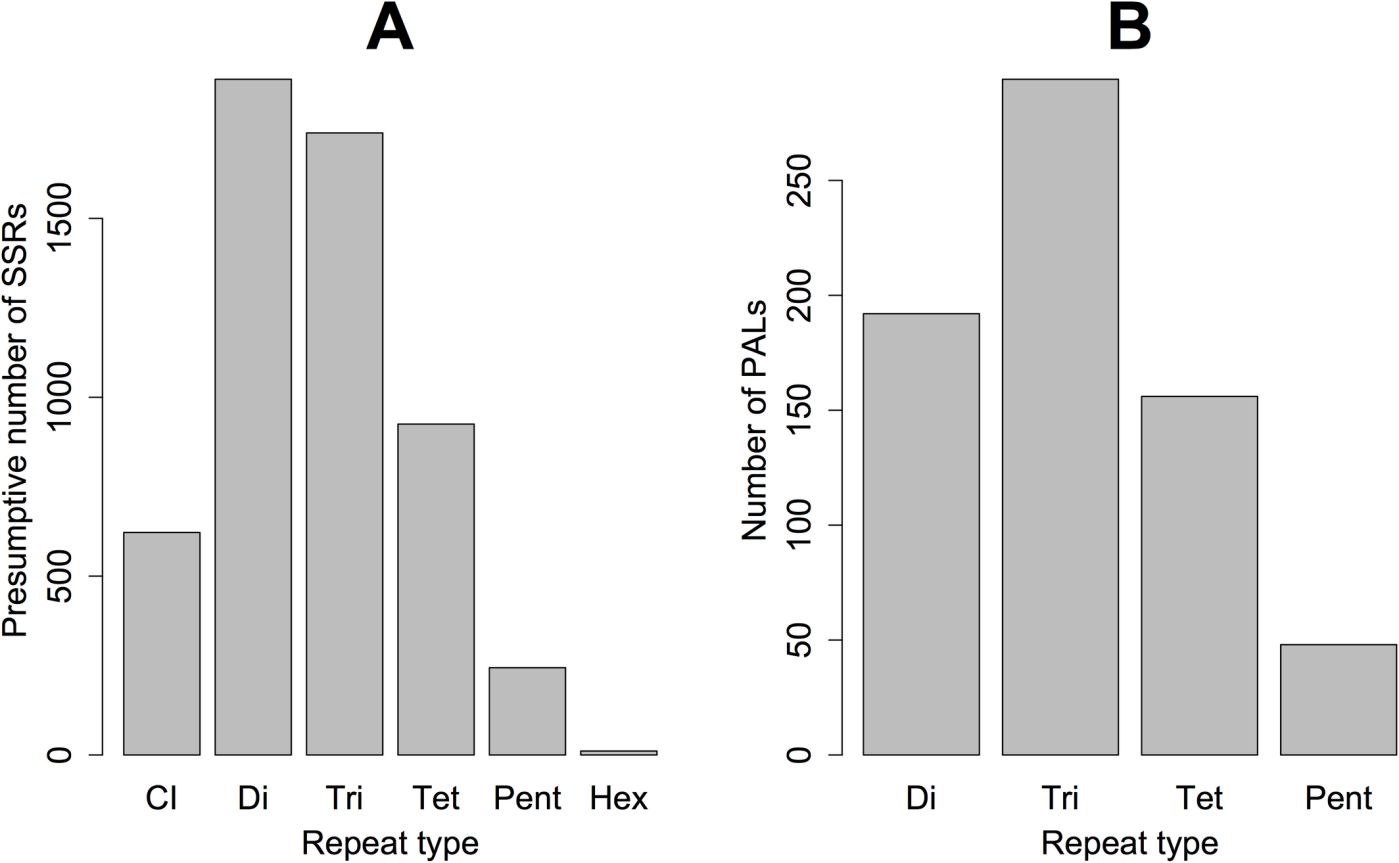
**The number of presumptive simple sequence repeat (SSR) loci (A) and potentially amplifiable microsatellite loci (PALs) for which primers were designed (B) identified using MSATCOMMANDER.** CI = compound/interrupted, di = dinucleotide, tri = trinucleotide, tet = tetranucleotide, pent = pentanucelotide, and hex = hexanucelotide.

In total, the 454 runs generated 113,739,428 bp of sequence across 283,830 reads (S2–S5 Files). Of these reads, 88,240 were generated via the XLR70 chemistry (mean length = 279.1 bp, St. dev. = 164.3 bp) and 195,590 were generated via the XL+ chemistry (mean length = 455.6 bp, St. dev. = 216.8 bp). MSATCOMMANDER identified a total of 5430 repeat containing fragments (approximately 1.9% of all reads), which are depicted by repeat class in Fig 2A. Of these fragments, 690 corresponded to tandemly repeated microsatellites with sufficient flanking sequence for primer design (*i*.*e*., are non-compound/non-interrupted PALs; Fig 2B). The results of BLASTn searches of NCBI’s nr/nt database using 454 fragments containing molecularly pursued PALs as queries are given in S1 Table.

### PAL screening

Of the 40 PALs selected for molecular screening (see S1 File for primer sequences) in WLU, we identified a set of 27 loci that exhibited a high rate of successful amplification (Table 2). The discrepancy among replicate genotypes was negligible for the vast majority of loci (min = 0%, max = 12.5%, mean = 0.4%, SD = 2.4%). In screening the SBI and OH/PA localities we identified a subset of 14 loci (Table 3) with a high rate of amplification success (87% across all loci). However, the majority of missing data were associated with *Pc12* and *Pc25*, where respective success rates equaled 17% and 50% among SBI and OH/PA localities. Consistent with the pattern observed in the larger set of loci that performed well in the WLU population, we observed a low genotyping error rate among replicate reactions from the SBI and OH/PA populations (min = 0%, max = 1.8%, mean = 0.6%, SD = 0.8%). The scored and binned microsatellite genotypes used to perform all subsequent analyses are given in S6 File.

**Table 2:** Genetic diversity indices and summary statistics for the 27 microsatellite loci that reliably amplified in samples from the Washington and Lee University locality.

Locus	N	No. Alleles	Obs. Het	Exp. Het	F_IS_	Allelic Richness	Effective Alleles
Pc4	31	13	0.871	0.804	-0.0672	13	5.098
Pc5	24	3	0.125	0.539	0.7767	2.33	2.169
Pc7	32	6	0.500	0.537	0.084	4.13	2.158
Pc8	32	6	0.500	0.524	0.0624	4.86	2.103
Pc9	20	6	0.150	0.751	0.8094	6	4.020
Pc10	20	6	0.200	0.718	0.7333	6	3.540
Pc11	28	4	0.429	0.573	0.2694	4	2.344
Pc12	25	2	0.160	0.147	-0.0667	1.14	1.173
Pc13	30	3	0.333	0.376	0.1304	2.7	1.603
Pc14	28	3	0.393	0.545	0.2962	2.45	2.199
Pc15	25	4	0.280	0.590	0.5397	3.36	2.437
Pc16	27	5	0.741	0.729	0.0029	4.32	3.691
Pc17	27	6	0.704	0.737	0.0635	5.31	3.797
Pc20	31	3	0.355	0.343	-0.0185	2.51	1.522
Pc21	27	2	0.370	0.483	0.2507	2	1.934
Pc22	31	3	0.194	0.180	-0.0619	3	1.219
Pc23	31	6	0.387	0.735	0.4857	6	3.769
Pc25	14	5	0.357	0.717	0.529	3.32	3.532
Pc26	23	5	0.087	0.578	0.8557	5	2.372
Pc27	27	4	0.370	0.642	0.4384	4	2.793
Pc28	31	4	0.742	0.634	-0.1538	3.77	2.734
Pc34	10	4	0.300	0.535	0.4808	4	2.151
Pc35	9	3	0.889	0.537	-0.6203	3	2.160
Pc36	30	1	0.000	0.000	NA	1	1.000
Pc37	32	9	0.813	0.713	-0.1241	6.19	3.483
Pc39	29	1	0.000	0.000	NA	1	1.000
Pc40	10	2	0.300	0.255	-0.125	2	1.342
**Mean**	25.3	4.407	0.391	0.516	0.223	3.940	2.494
**SEM**	1.363	0.484	0.049	0.044	0.073	0.458	0.204

**Table 3:** Diversity indices and summary statistics for the 14 loci that reliably amplified samples from Ohio and Pennsylvania localities.

Locus/Pop	N	No. Alleles	Obs. Het	Exp. Het	F_IS_	Allelic Richness	Effective Alleles
**South Bass Island**							
Pc3	10	1	0.000	0.000	NA	1.000	1.000
Pc5	12	3	0.417	0.344	-0.170	2.700	1.524
Pc7	12	3	0.250	0.344	0.313	2.820	1.524
Pc8	11	2	0.091	0.087	NA	1.910	1.095
Pc12	6	1	0.000	0.000	NA	1.000	1.000
Pc13	12	1	0.000	0.000	NA	1.000	1.000
Pc14	12	1	0.000	0.000	NA	1.000	1.000
Pc15	12	5	0.500	0.524	0.090	4.670	2.102
Pc16	12	1	0.000	0.000	NA	1.000	1.000
Pc17	12	1	0.000	0.000	NA	1.000	1.000
Pc20	11	2	0.091	0.087	NA	1.880	1.095
Pc25	7	3	0.714	0.602	-0.111	2.840	2.513
Pc28	10	3	0.500	0.535	0.118	3.000	2.151
Pc37	12	2	0.083	0.080	NA	1.820	1.087
Pop mean	10.787	2.071	0.189	0.186	0.048	1.974	1.364
SEM	0.490	0.300	0.061	0.058	0.071	0.276	0.130
**Squire Valleevue**							
Pc3	10	2	0.500	0.495	0.043	2.000	1.980
Pc5	8	9	0.750	0.852	0.185	9.000	6.737
Pc7	10	2	0.300	0.255	-0.125	2.000	1.342
Pc8	10	2	0.300	0.255	-0.125	2.000	1.342
Pc12	0	0	0.000	0.000	NA	0.000	0.000
Pc13	10	1	0.000	0.000	NA	1.000	1.000
Pc14	8	1	0.000	0.000	NA	1.000	1.000
Pc15	9	1	0.000	0.000	NA	1.000	1.000
Pc16	10	4	0.500	0.410	-0.169	4.000	1.695
Pc17	9	3	0.444	0.549	0.247	3.000	2.219
Pc20	10	3	0.600	0.485	-0.187	3.000	1.942
Pc25	4	5	0.500	0.688	0.400	5.000	3.200
Pc28	10	3	0.300	0.395	0.290	3.000	1.653
Pc37	10	3	0.700	0.605	-0.105	3.000	2.532
Pop mean	8.429	2.786	0.350	0.356	0.045	2.786	1.974
SEM	0.782	0.595	0.071	0.075	0.069	0.595	0.421
**Doan Brook**							
Pc3	11	2	0.364	0.298	-0.177	2.000	1.424
Pc5	12	7	0.500	0.781	0.397	5.860	4.571
Pc7	12	3	0.417	0.344	-0.170	2.830	1.524
Pc8	12	2	0.417	0.413	0.035	2.000	1.704
Pc12	1	1	0.000	0.000	NA	1.000	1.000
Pc13	12	1	0.000	0.000	NA	1.000	1.000
Pc14	10	1	0.000	0.000	NA	1.000	1.000
Pc15	11	5	0.364	0.388	0.111	4.410	1.635
Pc16	11	4	0.727	0.591	-0.185	3.910	2.444
Pc17	12	3	0.583	0.569	0.019	3.000	2.323
Pc20	12	3	0.417	0.344	-0.170	2.830	1.524
Pc25	7	5	0.143	0.765	0.838	3.530	4.261
Pc28	12	3	0.417	0.497	0.203	2.820	1.986
Pc37	12	4	0.583	0.656	0.154	3.840	2.909
Pop mean	10.500	3.143	0.352	0.403	0.096	2.859	2.093
SEM	0.817	0.467	0.062	0.071	0.093	0.376	0.303
**Manatoc Boy Scout Camp**							
Pc3	24	2	0.333	0.413	0.214	2.000	1.704
Pc5	23	10	0.652	0.852	0.255	7.040	6.739
Pc7	24	3	0.458	0.374	-0.205	2.840	1.598
Pc8	24	4	0.583	0.544	-0.051	3.220	2.194
Pc12	0	0	0.000	0.000	NA	0.000	0.000
Pc13	24	1	0.000	0.000	NA	1.000	1.000
Pc14	18	1	0.000	0.000	NA	1.000	1.000
Pc15	22	10	0.545	0.691	0.233	6.320	3.237
Pc16	24	8	0.625	0.769	0.208	5.970	4.331
Pc17	24	5	0.792	0.732	-0.061	4.760	3.728
Pc20	23	2	0.261	0.287	0.114	1.990	1.403
Pc25	10	12	0.800	0.875	0.138	6.200	8.000
Pc28	24	5	0.417	0.559	0.274	3.240	2.268
Pc37	24	4	0.417	0.438	0.071	3.520	1.781
Pop mean	20.571	4.786	0.420	0.467	0.108	3.507	2.784
SEM	1.889	1.017	0.074	0.083	0.047	0.599	0.605
**Allegheny National Forest**							
Pc3	11	2	0.545	0.397	-0.333	2.000	1.658
Pc5	10	7	0.600	0.730	0.229	6.190	3.704
Pc7	11	2	0.273	0.236	-0.111	2.000	1.308
Pc8	11	2	0.455	0.483	0.107	2.000	1.936
Pc12	2	1	0.000	0.000	NA	1.000	1.000
Pc13	11	1	0.000	0.000	NA	1.000	1.000
Pc14	7	1	0.000	0.000	NA	1.000	1.000
Pc15	10	5	0.600	0.725	0.223	4.910	3.636
Pc16	10	6	0.700	0.700	0.053	6.000	3.333
Pc17	11	3	0.364	0.314	-0.111	2.960	1.458
Pc20	11	3	0.455	0.368	-0.191	2.900	1.582
Pc25	6	7	0.833	0.764	0.000	5.230	4.235
Pc28	11	3	0.182	0.169	-0.026	2.810	1.204
Pc37	10	3	0.500	0.405	-0.184	3.000	1.681
Pop mean	9.429	3.286	0.393	0.378	-0.031	3.071	2.052
SEM	0.709	0.569	0.072	0.074	0.050	0.485	0.307

### Quality control and summary statistics

Sample sizes, diversity statistics, and inbreeding coefficients for the WLU population are given in Table 2. Upon applying Holm’s ([62]; FWER = 0.05) correction for multiple testing by treating each population as a family of tests, seven loci significantly deviated from Hardy-Weinberg proportions in WLU (*Pc5*, *Pc9*, *Pc10*, *Pc15*, *Pc23*, *Pc25*, and *Pc26*). In addition to detecting evidence of null alleles at these loci, MICRO-CHECKER also detected evidence of null alleles at *Pc27*, and *Pc34*. There was no evidence of genotypic disequilibrium among any pair of loci in WLU after correcting for multiple testing [62].

Sample sizes, diversity statistics, and inbreeding coefficients are given for SBI and the OH/PA sites in Table 3. Among SBI and the OH/PA locales, only one significant deviation from Hardy-Weinberg proportions was detected following adjustments for multiple testing via Holm’s [62] correction (*Pc25* in DB). Evidence for null alleles was detected at *Pc5* in DB and MBSC, and at *Pc15* in MBSC. There was no statistical evidence for genotypic disequilibrium among pairs of loci within or across OH/PA locales upon correcting for multiple testing [62].

### Bottleneck tests and effective population size estimates

We did not find consistent evidence across methods (*i*.*e*., heterozygosity excess tests and *M*-ratios) for recent population reductions at any of our sites (Table 4). However, we did obtain significant Wilcoxon tests for WLU under the IAM and TPM. We also obtained mean *M*-ratios < 0.68 in SQ and ANF. NeESTIMATOR was unable to produce point estimates of and/or confidence limits for N_e_ at all of our sites (S2 Table). However, the information we obtained from NeESTIMATOR is consistent with the idea that SBI has a smaller effective population size (point estimate = 65) than WLU (lower confidence limit = 153.4) and several of the mainland OH/PA sites (point estimate for MBSC = 517.8 and point estimate for ANF = 158.3).

**Table 4:** Results of heterozygosity excess tests and *M*-ratios for population bottleneck detection.

Population	Mutation Model	Wilcoxon Test	Mode-shift Test	*M*-Ratio (SEM)
Washington and Lee University				
	IAM	**0.003**	L—shaped	0.740 (0.044)
	SMM	0.933		
	TPM	**0.018**		
South Bass Island				
	IAM	0.988	L–shaped	0.800 (0.074)
	SMM	0.996		
	TPM	0.988		
Squire Valleevue				
	IAM	0.150	L-shaped	**0.649** (0.087)
	SMM	0.674		
	TPM	0.213		
Doan Brook				
	IAM	0.161	L—shaped	0.683 (0.094)
	SMM	0.784		
	TPM	0.278		
Manatoc Boy Scout Camp				
	IAM	0.080	L—shaped	0.699 (0.077)
	SMM	0.919		
	TPM	0.138		
Allegheny National Forest				
	IAM	0.652	L—shaped	**0.615** (0.079)
	SMM	0.687		
	TPM	0.794		

Heterozygosity excess tests were investigated under the infinite alleles model (IAM), stepwise mutation model (SMM), and two-phase mutation model (TPM). The probabilities reported for Wilcoxon signed-rank tests correspond to a one-tailed test of heterozygosity excess. The mean *M*-ratio across loci for each population was assessed against a critical value of 0.68.

### Detection of outlier loci

As shown in S1 File, only Pc13 exhibited any evidence of being an outlier. However, this result was not statistically significant after adjusting for multiple testing (FDR = 0.05). Consequently, in what follows, we assume that all of the loci used in our analyses of population differentiation are at least passably neutral.

### Population differentiation

Analyses that examined genetic differentiation between all sampled locales were based on the 11 loci (*Pc5*, *Pc7*, *Pc8*, *Pc13*, *Pc14*, *Pc15*, *Pc16*, *Pc17*, *Pc20*, *Pc28*, *Pc37*; see above) that reliably amplified in all populations. We excluded *Pc12* and *Pc25* due to complete failure within some OH/PA localities and *Pc3* for failure in WLU. We opted to include *Pc13* and *Pc14* when comparing all six locales as these two loci were polymorphic in WLU. However, we removed *Pc13* and *Pc14* for our investigations of fine-scale population differentiation among OH/PA locales as these markers were monomorphic across all of these sites.

Locus-specific estimates of G_ST_ across all six populations ranged from 0.215–0.762, and were highly statistically significant (maximum *P*-value = 0.0001). Locus-specific values of G"_ST_ ranged from 0.524–0.883 and were also all highly statistically significant (maximum *P*-value = 0.0001). Combining information across all loci, the global estimate of G_ST_ was 0.351 (SE = 0.034, *P-*value = 0.0001) and the analogous estimate for G"_ST_ was 0.669 (SE = 0.044, *P-*value = 0.0001). Pairwise comparisons revealed pronounced differentiation between WLU and all other sampled localities (Table 5). Interestingly, we observed similar degrees of differentiation between SBI vs. OH/PA locales and WLU vs. OH/PA locales.

**Table 5:** Pairwise estimates of G_ST_ and G"_ST_ based on the 11 loci that reliably amplified samples from all locales.

**G**_**ST**_	SBI	SQ	ANF	DB	MBSC
SBI	*	0.0001	0.0001	0.0001	0.0001
SQ	0.441	*	0.0001	0.594	0.0001
ANF	0.420	0.114	*	0.0001	0.0001
DB	0.390	-0.002	0.081	*	0.0001
MBSC	0.358	0.075	0.148	0.065	*
WLU	0.400	0.277	0.295	0.252	0.208
**G**"_**ST**_	SBI	SQ	ANF	DB	MBSC
SBI	*	0.0001	0.0001	0.0001	0.0001
SQ	0.848	*	0.0001	0.529	0.0001
ANF	0.837	0.331	*	0.0001	0.0001
DB	0.817	-0.006	0.257	*	0.0001
MBSC	0.801	0.243	0.463	0.227	*
WLU	0.936	0.830	0.896	0.822	0.740

Values of both G-statistics are located below the diagonal with corresponding *P*-values above. All significance tests performed in GENALEX are based on 9,999 permutations, and signify P(permuted ≥ observed). All pairwise comparisons were statistically significant with the exception of DB and SQ.

**Fig 3:**
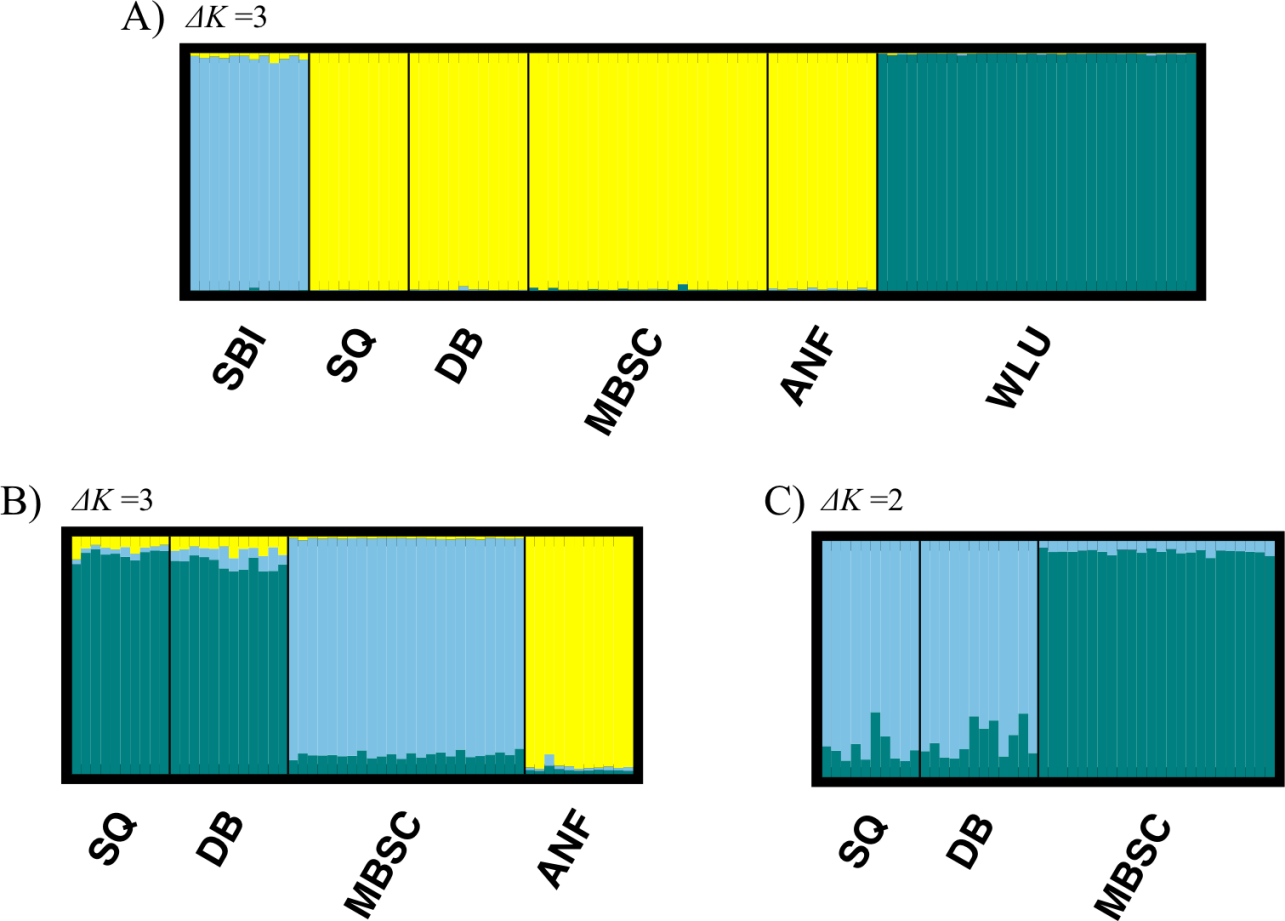
Results from STRUCTURE analyses. (A) Population structuring among the six localities following the optimal solution of ΔK = 3 using 11 microsatellite loci. (B) Population structuring among mainland Ohio and Pennsylvania localities revealed the optimal number of clusters to be ΔK = 3 across 9 loci. (C) Population structuring among only mainland Ohio localities revealed the optimal number of genetic clusters to be ΔK = 2.

The large-scale STRUCTURE analysis suggested K = 3 as the optimal value of K (S1 Fig; S2 Fig). The WLU and SBI localities were identified as unique genetic clusters while the mainland OH/PA locales were assigned to a single cluster. Not surprisingly, there was no evidence for admixture detected among the three genetic clusters identified (Fig 3A). The AMOVA performed on these three genetic clusters revealed that differences among clusters explained over one-third of the total variance (Table 6).

**Table 6:** AMOVA results based on the three genetic clusters identified from the large-scale analysis performed in STRUCTURE.

Source of Variation	Variation Explained	Degrees of Freedom	Sum of Squares	Variance Component	Fixation Index	*P*-value^a^
Among clusters	37%	2	209.887	1.766	F_ST_ = 0.374	0.0001
Among individuals	18%	98	371.569	0.836	F_IS_ = 0.283	0.0001
Within individuals	45%	101	214.000	2.119	F_IT_ = 0.551	0.0001
Total	100%	201	795.455	4.721	NA	NA

^**a**^All significance tests performed in GENALEX based on 9,999 permutations, and signify P(permuted ≥ observed) for all fixation indices reported.

Results from the STRUCTURE runs that investigated fine-scale population differentiation among OH/PA populations suggested K = 3 as the optimal value for K when all four OH/PA sites were considered (S3 Fig; S4 Fig; Fig 3B). In this analysis, ANF was identified as a well-defined cluster and all ANF individuals were unambiguously assigned to this cluster. Among the remaining three mainland OH sites, SQ and DB formed a cluster and MBSC formed the third cluster. While membership in the SQ/DB and MBSC clusters is also well-defined, it is considerably ‘fuzzier’ than ANF cluster membership (Fig 3B). In particular, individuals assigned to the MBSC cluster showed substantial and consistent admixture proportions with the SQ/DB cluster. To further address patterns of differentiation among SQ, DB, and MBSC, we conducted a third analysis in STRUCTURE that only considered these three sites (Fig 3C). In this analysis, K = 2 was the optimal solution (S5 Fig; S6 Fig) with one cluster corresponding to MBSC and the other cluster corresponding to SQ/DB. As shown in Fig 3C, this analysis suggests substantive admixture between MBSC and SQ/DB.

**Fig 4:**
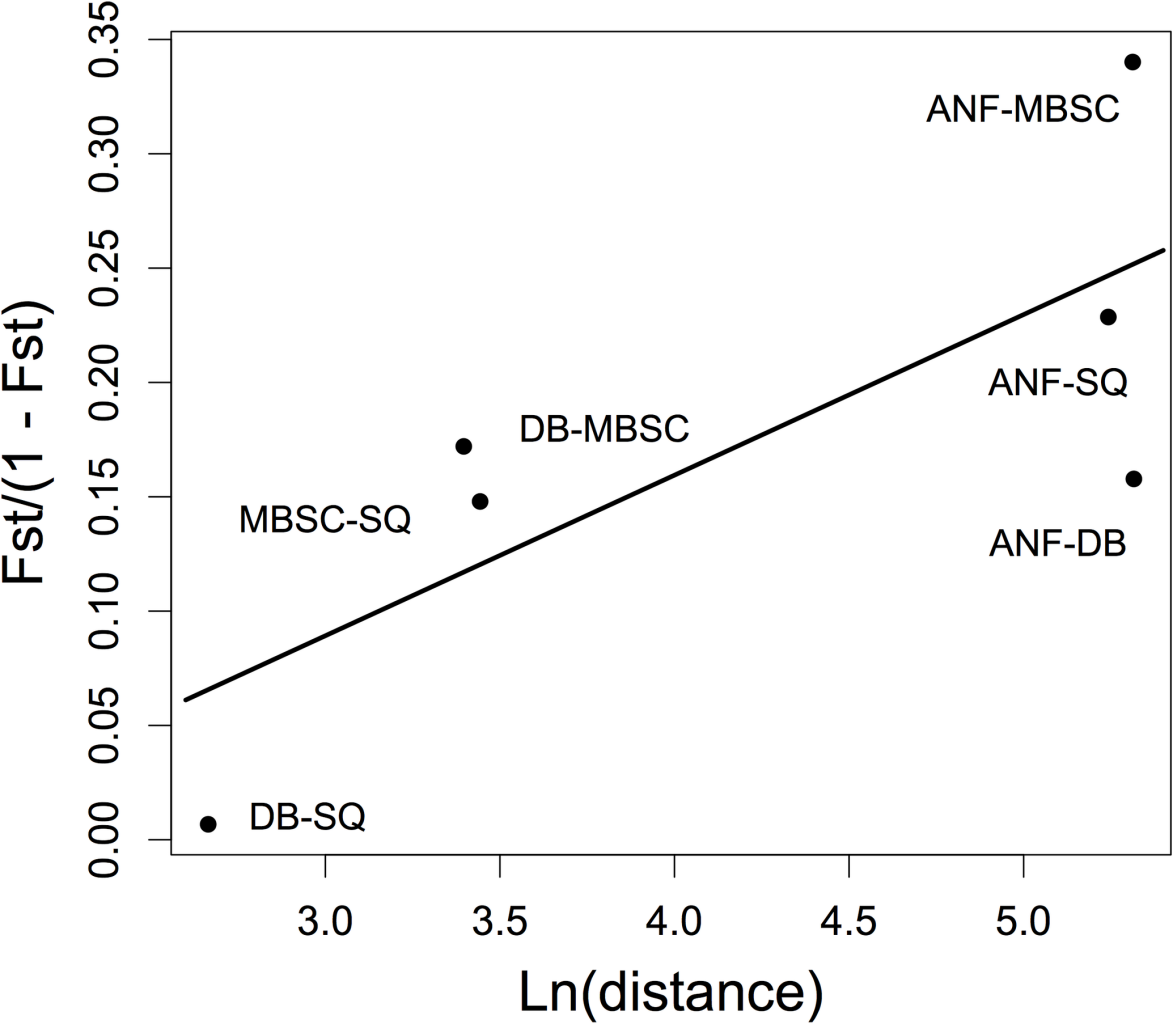
Relationship between geographic distance and genetic differentiation. Regressing linearized F_ST_ values against the natural logarithm of distance (km) revealed a positive relationship indicative of IBD among sites within the OH/PA cluster.

In order to examine the degree of IBD among sites within the OH/PA cluster, we also regressed F_ST_/(1 –F_ST_) against the natural logarithm of distance between sites in km. The equation estimated by this regression is *y* = -0.122 + 0.070*x* with approximate 95% confidence intervals of -0.376–0.069 for the intercept and 0.024–0.157 for the slope, suggesting the effects of isolation by distance within the OH/PA cluster are not negligible. Nevertheless, as can be seen in Fig 4 the results of this analysis are generally congruent with the small-scale analyses we performed in STRUCTURE (Fig 3B and 3C).

**Fig 5:**
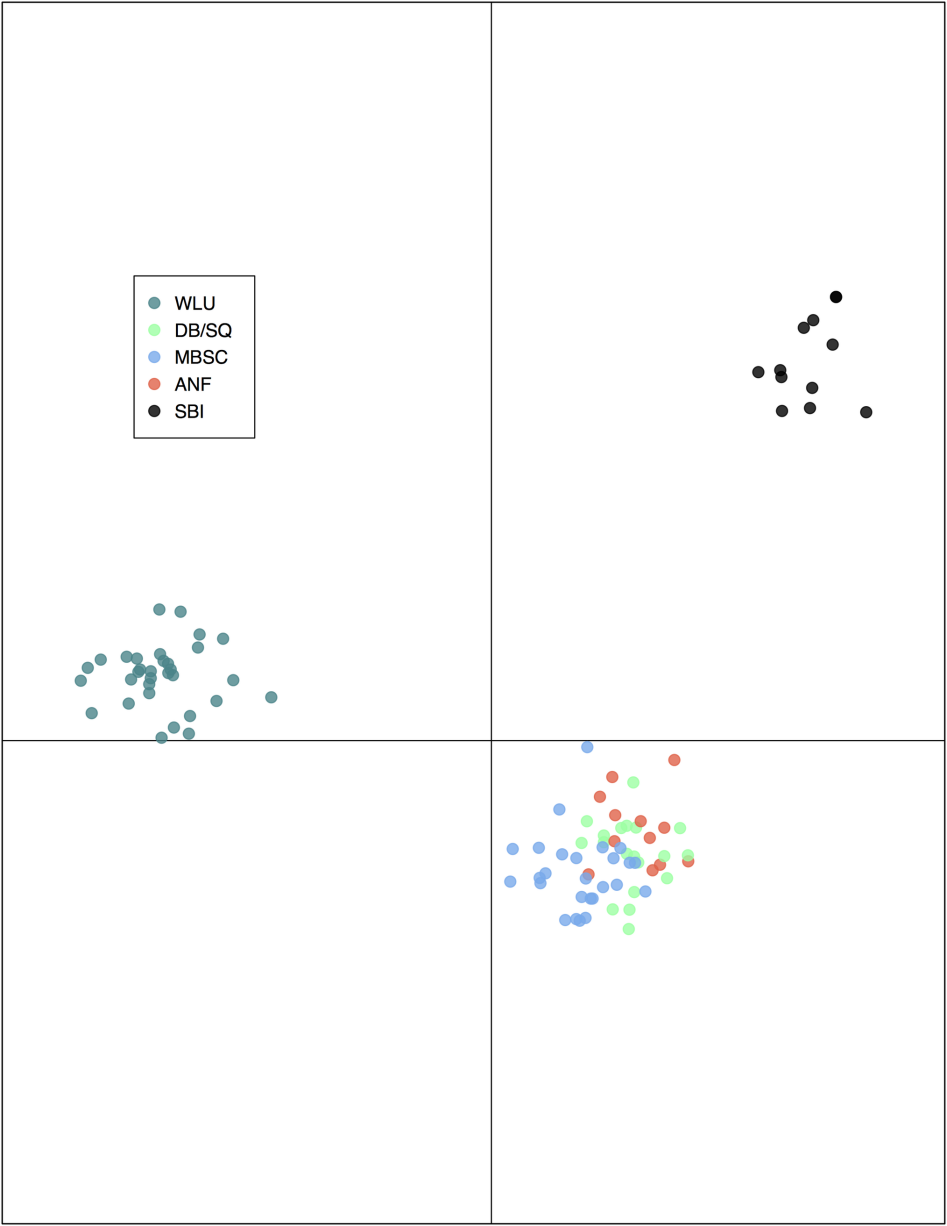
DAPC scatter plot. Scatter plot of the first two discriminant functions revealed groupings consistent with the results from STRUCTURE. The first discriminant function identifies three clusters: WLU, SBI, and mainland OH/PA. The second discriminant function further separates the three major clusters, SBI in particular, while also providing some separation within the mainland OH/PA cluster.

K-means clustering and BIC suggested that K = 4–7 all represented essentially equally valid summaries of our data (S7 Fig). As such, we chose to focus on K = 5 when conducting DAPC, as we found this solution to be the most straightforward value of K to interpret. The cross-validation procedure determined that 10 principal components was the optimal value to retain and no discriminant functions were discarded. The five clusters strongly corresponded to locales as follows: (1) “ANF cluster” (11 ANF individuals and 1 DB individual), (2) “WLU cluster” (32 WLU individuals), (3) “MBSC cluster” (23 MBSC individuals, 2 DB individuals, and 1 SQ individual), (4) “SQ/DB cluster” (9 SQ individuals, 9 DB individuals, and 1 MBSC individual), and (5) “SBI cluster” (12 SBI individuals). As can be seen in S8 Fig, cluster membership probabilities were generally high and there were no cases in which cluster assignments were marginal. Scatter plots of the first two discriminant functions revealed that the first discriminant function separates the same three groups identified using STRUCTURE: WLU, SBI, and mainland OH/PA (Fig 5). The second discriminant function further distinguishes among the three major groups, while also providing some separation between the “MBSC” and “ANF” clusters.

## Discussion

### Overview

In this study, we used 454 pyrosequencing to identify hundreds of potentially amplifiable microsatellite loci from the red-backed salamander—an ecologically important species that has long-served as a model system in evolutionary ecology [2–19, 21–27]. In addition, we molecularly screened 40 of our top candidate PALs in six populations that are geographically removed from each other by a variety of scales ranging from ~ 10–500 km. This enabled us to identify loci that were informative in samples from Virginia, Pennsylvania, and Ohio—including an isolated island population (SBI). Ultimately, we used these loci to conduct surveys of neutral variation in all of the populations that we sampled and to examine differentiation among these locales. The results of these analyses are consistent with the idea that SBI has lower genetic diversity than our other sites, presumably due to a founder’s effect. In addition, our analyses show that WLU is markedly differentiated from OH/PA and that SBI is markedly differentiated from WLU and OH/PA. In what follows, we discuss the utility of the genetic resources that we have discovered and patterns of variation within and between populations.

### Utility of new genetic resources

Attempts to utilize microsatellite markers developed from large, well-studied populations in western Virginia [20] in other parts of the red-backed salamander’s range have varied in success from none [13] to loss of a small number of screened loci [17,21] to 100% informative markers [22]. However, to the best of our knowledge, all studies of the red-backed salamander that have used microsatellites have been restricted to a relatively small number of loci ≤ 7 [13–19,21–23]. By screening the markers that we developed in three highly divergent regions of the red-backed salamander’s range, we were able to identify 27 new markers for use in the well-studied region of western Virginia. Moreover, we identified 14 new markers for use in western Pennsylvania and northeastern Ohio—a part of the range that is becoming increasingly well studied from an ecological perspective (see [63] and citations within). Finally, we identified 11 markers that enabled informative analyses of population differentiation between locales in Virginia, western Pennsylvania/northeastern Ohio, and an isolated island population ~ 4.5 km off the shore of Lake Erie. These new markers allowed us to correctly assign 100% of the individuals we sampled to their geographic region of origin (Figs 3 and 5). In addition, small-scale STRUCTURE analyses within the OH/PA cluster correctly assigned 100% of ANF and MBSC individuals to their locales of origin and 100% of SQ and DB individuals to a cluster that encompassed these two undifferentiated locales (pairwise G"_ST_ = -0.006). Similarly, DAPC results based on these markers recovered analogous clusters to those obtained from STRUCTURE and yielded similar albeit slightly weaker patterns of correct individual assignment. Collectively, these results show that the panel of markers we have identified are informative at relatively small spatial scales, even in comparisons involving populations that were founded as a result of post-glacial range expansion (see below). While our endeavors will not eliminate the need for researchers working in other parts of the range to conduct screens to verify amplification success and polymorphism in their study populations, they do provide meaningful guidance about which loci to try first. Indeed, ongoing work in one of our labs (RBP) has begun to extend a subset of these markers (*Pc3*, *Pc4*, *Pc5*, *Pc7*, *Pc8*, *Pc15*, *Pc16*, *Pc17*, *Pc20*, *Pc22*, *Pc28*, *Pc34*, *Pc37*, *Pc38*, *Pc39*, and *Pc40*) to the Peaks of Otter Salamander, *Plethodon hubrichti*, and we recommend starting with this panel of 16 markers when transferring markers to unstudied red-backed salamander populations and cross-amplifying markers in closely related species.

### Genetic variation within populations

#### South bass island

The most striking pattern when comparing intra-population diversity and summary statistics across populations is how much lower diversity estimates from SBI are relative to the other five locales we sampled. For example, in SBI, 6 of 14 (42.9%) loci were monomorphic in comparison to values ranging from 2 of 27 (7.4%; WLU) to 3 of 13 (23.1%; SQ) at other sites (these calculations do not consider “monomorphic” loci at which < 5 individuals were genotyped). Moreover, mean A_R_ for SBI was 1.974, whereas among OH/PA sites mean A_R_ varied between 2.786–3.507 and mean A_R_ at WLU was 3.940. These findings are likely a consequence of a founder’s effect followed by strong drift in the SBI population. However, we were unable to detect other genetic signatures of a recent bottleneck in SBI, and this population appears to be more-or-less in Hardy-Weinberg equilibrium. Given our fairly small sample size in SBI (average across loci ~ 11 individuals) and the tendency of bottleneck tests to be underpowered [64], it is possible that failure to explicitly detect evidence of a recent bottleneck in SBI is a Type II Statistical Error.

#### Northeastern Ohio and western Pennsylvania

Among mainland OH/PA sites, ANF is within a large tract of continuous forest and MBSC is within a considerable, albeit occasionally interrupted, wooded area near the shores of a small manmade lake. On the other hand, SQ is within a somewhat isolated patch of woods that is surrounded by farmland and suburban residential areas, while DB is within a ~ 2 km strip of woods (< 200 m wide) that is surrounded on both sides by urban residential areas. MBSC had the highest mean A_R_ (3.507), followed by ANF (3.071), DB (2.859), and SQ (2.786). However, other measures of diversity (*e*.*g*., H_e_ and effective number of alleles) did not follow this pattern. Thus, as reported by Jordan et al. [22] for sites in northeastern Indiana, there does not appear to be a clear positive relationship between habitat patch size and genetic diversity among OH/PA sites. Because NeESTIMATOR was unable to compute point estimates of N_e_ for SQ and DB, we could not directly assess the relationship between patch size and effective population size. However, the sampling effort required to collect individuals at DB was substantially lower than SQ, which suggests a larger census population size within DB. Differences in salamander abundance between DB and SQ probably reflect the profusion of high-quality microhabitat (rocks) at DB. As was the case with SBI, we did not detect unequivocal genetic signatures of recent population reductions at any of the OH/PA sites. However, SQ and ANF both had mean M-ratios < 0.68, although these estimates were within one SE of this critical value. None of the OH/PA sites were markedly out of Hardy-Weinberg equilibrium, with DB, MBSC, and SQ having mild homozygote excess (on average ~ 5% to < 1% more homozygosity than expected) and ANF exhibiting mild heterozygote excess (on average 1.5% less homozygosity than expected). Collectively, these results suggest that these mainland OH/PA populations are not highly inbred even though some of them (SQ and DB) are restricted to small isolated patches of habitat.

#### Washington and Lee University

The WLU site is within an isolated stand of trees approximately 0.55 km^2^ in total area that is boarded by the city of Lexington, Virginia to the south and east, pastureland to the west and northwest, and the Maury River to the north and northeast. A_R_ at this site was somewhat higher than at the other sites in our study, and it is possible that this is related to the older age of Appalachian populations relative to sites that were glaciated during the Pleistocene [22,24]. However, the mean inbreeding coefficient for WLU was moderately large (F_IS_ = 0.223, SE = 0.073), and while this may be attributable to null alleles, there is evidence that the WLU population has undergone a recent population reduction, as heterozygosity excess tests were significant under the IAM and TPM. Interestingly, surveys of dinucleotide loci from sites within large tracts of continuous forest near Mountain Lake Biological Station (37.375531, 80.523235; ~ 100 km straight line distance from WLU) resulted in considerably larger genetic richness estimates (on average 7–10 alleles per locus depending on the locale; [14,15]) than those reported here. Although these comparisons may not be straightforward due to differences between the mutation rates of dinucleotide vs. tri and tetranucelotide repeats [65], they suggest that the WLU population may have reduced neutral diversity relative to populations in undisturbed habitat within the same geographic region.

### Genetic variation between populations

#### General pattern

The pattern of genetic structuring recovered by our analyses of between population variation is one in which there is isolation by distance at small to intermediate spatial scales (~ 10–200 km) and marked differentiation among geographic regions. In a general sense, this finding is consistent with the original geographic patterns of protein electrophoretic rates offered by Highton and Webster [24]. However, the discovery of marked differentiation between mainland Ohio sites (DB, MBSC, and SQ) and SBI (pair-wise G_ST_ 0.358–0.441) demonstrates that there are isolated populations within the formerly glaciated portion of the red-backed salamander’s range that are very different from populations at relatively nearby locales. Similarly, Noël et al. [21] observed substantial differentiation between isolated populations in Montreal and populations in un-fragmented habitat approximately 190 km away in Mount Megantic National Park. Taken together, these results suggest that within the formerly glaciated portion of the red-backed salamander’s range, well differentiated isolated populations are not uncommon (see below for further discussion).

#### Levels of differentiation in un-glaciated vs. formerly glaciated regions

In a survey of six dinucleotide microsatellite loci across 10 sites (maximum distance between sites of 70 km) within the fragmented, rural landscape of northeastern Indiana, Jordan et al. [22] observed low but statistically significant levels of differentiation for 48% of pair-wise comparisons. In contrast, working in contiguous habitat in Giles County Virginia, Cabe et al. [14] reported low but statistically detectable levels of differentiation for over 80% of pair-wise comparisons between 50 m^2^ plots separated by distances of 2 km or less. While comparison of these studies is confounded with any differential effects that may exist between urban and rural landscapes, it suggests that the degree of differentiation among formerly glaciated sites in Ohio is more similar to formerly glaciated sites in Indiana than it is to un-glaciated sites in Virginia. Interestingly, in contrast to the results of Cabe et al. [14], Noël et al. [21] were unable to detect statistically significant differentiation among four sites within the continuous habitat of Mount Megantic National Park, Quebec that were separated by 0.8–4.1 km. As a whole, these results are consistent with the notion that differentiation among red-backed salamander populations is less pronounced in the formerly glaciated portion of the range than in the portion of the range that was never glaciated (see [21,24] for additional discussion). Further insight regarding levels and patterns of differentiation among populations within formerly glaciated regions will likely be gained from phylogeographic analysis, and recent work has indeed identified multiple lineages corresponding to patterns of post-glacial range expansion [66]. Because selection often acts to increase investment in dispersal at expanding range fronts [67], it is possible that descendants of ancestral populations which colonized formerly glaciated regions possess greater dispersal ability relative to descendants of ancestral populations that never left glacial refugia.

#### Degree of isolation among island populations

One of the more striking results of our study was that the degree of differentiation between SBI and Cleveland area populations ~ 100 km away (SB, DQ, MBSC), was greater than the degree of differentiation between these three sites and WLU. Indeed, the DAPC we performed ordinated OH/PA closer to WLU than SBI (Fig 5) despite the fact that WLU is > 400 km from any of the OH/PA sites. In comparisons between Long Island populations and mainland populations in Connecticut, New Jersey, and New York that were removed by distances ranging from 16–275 km, Fisher-Reid et al. [13] observed moderate to marked levels of differentiation depending on the pair of locales under consideration. However, in comparisons between Ile-Bizard, Ile-Perrot, and several sites on Montreal Island, Noël and Lapointe [17] observed marked differentiation despite the fact that none of their locales were separated by more than 50 km. Given that none of these islands (*i*.*e*., South Bass Island, Long Island, Ile-Bizar, Ile-Perrot, and Montreal Island) are particularly remote, these results strongly suggest that aquatic barriers as small as one km across, and perhaps even less, are capable of effectively isolating red-backed salamander populations [17]. This conclusion is further supported by ecological studies in Virginia that have shown second order streams reduce movement by approximately 50% and contribute to fine-scale genetic structuring [15].

## Conclusion

In this paper, we have presented genetic resources that will enable the scientific community to conduct population genetic studies in regions of the red-backed salamander’s range that have not previously been investigated in this manner. In addition, we have demonstrated the utility of these resources by using them to assess genetic variation within and between three well differentiated portions of this species range. In many respects, our findings are consistent with the original description of geographic patterns of protein variation for this species [24]. However, our results and the findings of others [17, 21] indicate that well differentiated isolated populations are not uncommon within formerly glaciated parts of the range.

## Supporting information

S1 Fig Mean LnP(D) plot for large-scale STRUCTURE run. (PDF)

S2 Fig Delta K plot for large-scale STRUCTURE run. (PDF)

S3 Fig Mean LnP(D) plot for OH/PA STRUCTURE run. (PDF)

S4 Fig Delta K plot for mainland OH/PA STRUCTURE run. (PDF)

S5 Fig Mean LnP(D) plot for mainland OH STRUCTURE run. (PDF)

S6 Fig Delta K plot for mainland OH STRUCTURE run. (PDF)

S7 Fig BIC values from K-means clustering solutions. (PDF)

S8 Fig DAPC cluster membership probability bar plot. Individuals are represented by a single bar. The number of clusters is K = 5 and colors correspond to those in Fig 5.
(PDF)

S1 File Primer sequences and results from outlier locus detection scans. (XLSX)

S2 File Sequences generated using Roche 454 pyrosequencing. (FASTA)

S3 File Sequences generated using Roche 454 pyrosequencing. (FASTA)

S4 File Sequences generated using Roche 454 pyrosequencing. (FASTA)

S5 File Sequences for the forty 454 fragments that contained molecularly pursued microsatellites. (FASTA)

S6 File Excel workbook containing scored and binned microsatellite genotypes. (XLSX)

S1 Table Results of BLASTn searches against NCBI’s nr/nt database using the forty 454 fragments that contained molecularly pursued PALs as queries. (DOCX)

S2 Table Effective population size estimates for the six *P*. *cinereus* populations. Ninety-five percent confidence intervals are given in parentheses. The symbol ∞ indicates that NeESTIMATOR was unable to estimate N_e_ and/or associated confidence limits.
(DOCX)
