# Supplementary figures and images for: Assessment of intra and interregional genetic variation in the Eastern Red-backed Salamander, Plethodon cinereus, via analysis of novel microsatellite markers
Source: PLoS One. 2017 Oct 20;12(10):e0186866. doi: 10.1371/journal.pone.0186866 (PMC5650168; doi:10.1371/journal.pone.0186866)

L(K) (mean  $\pm$  SD)

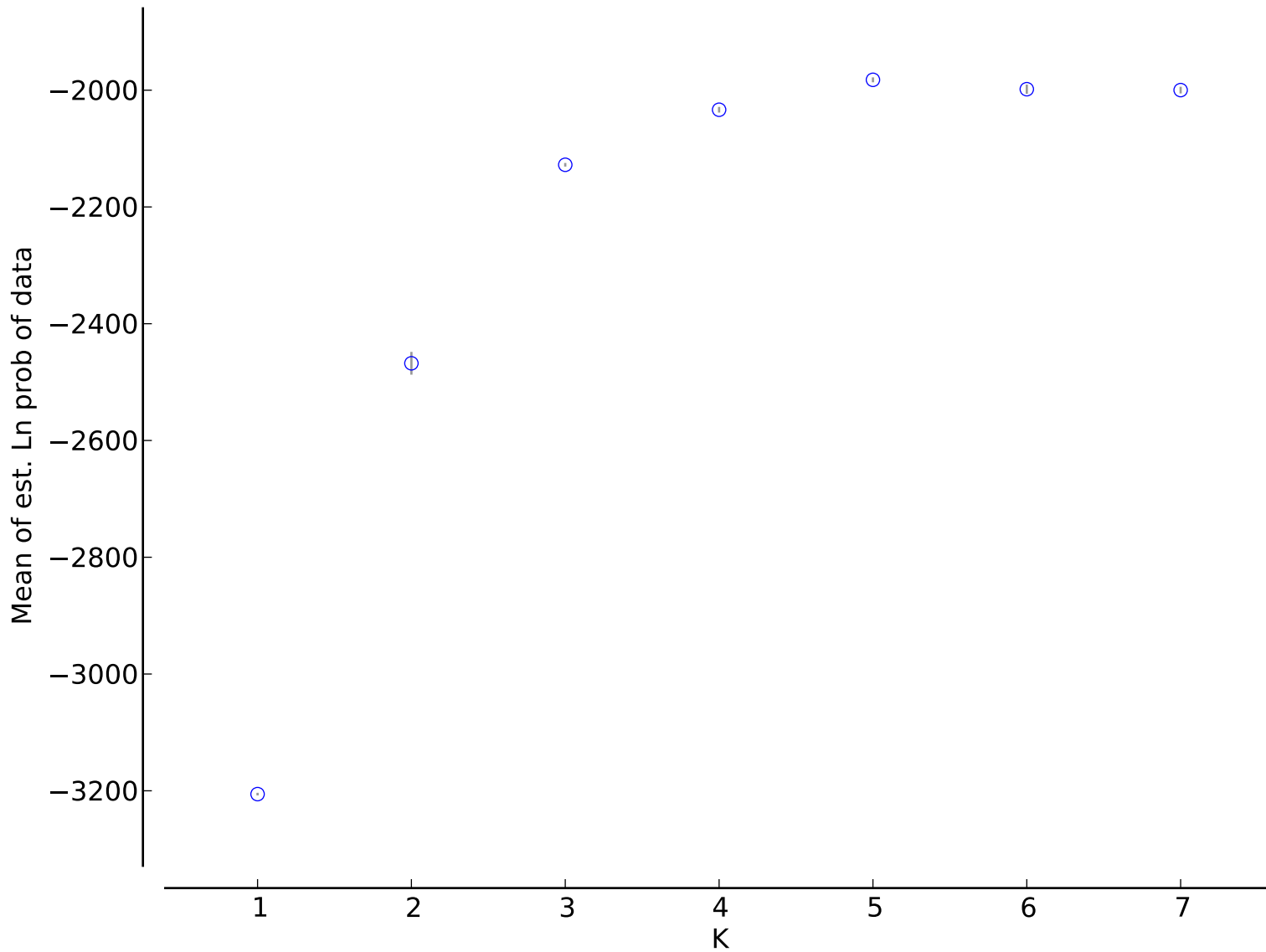

Supplement: S1 Fig — (PDF) [file pone.0186866.s001.pdf]

$$\text{DeltaK} = \text{mean}(|L''(K)|) / \text{sd}(L(K))$$

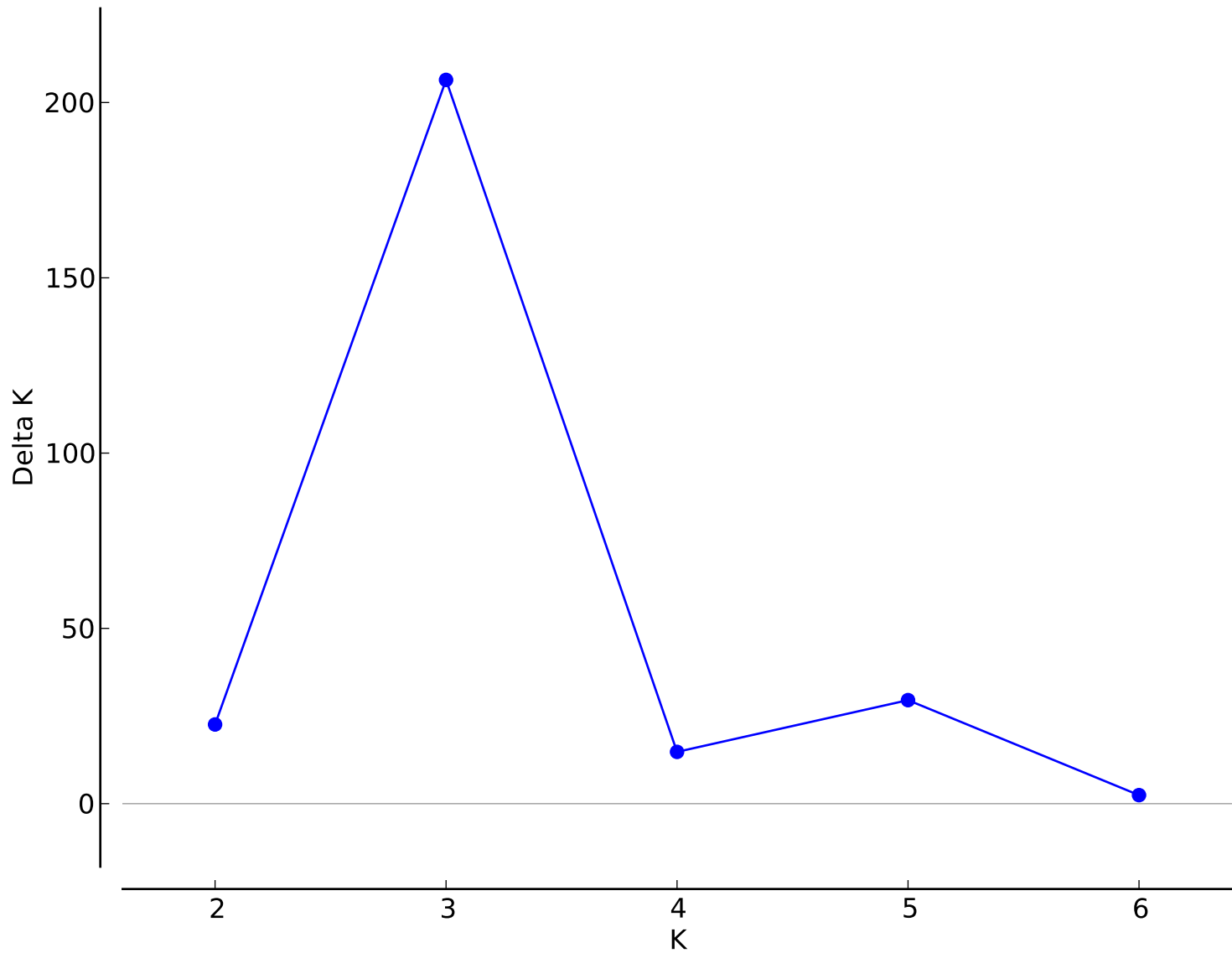

Supplement: S2 Fig — (PDF) [file pone.0186866.s002.pdf]

L(K) (mean  $\pm$  SD)

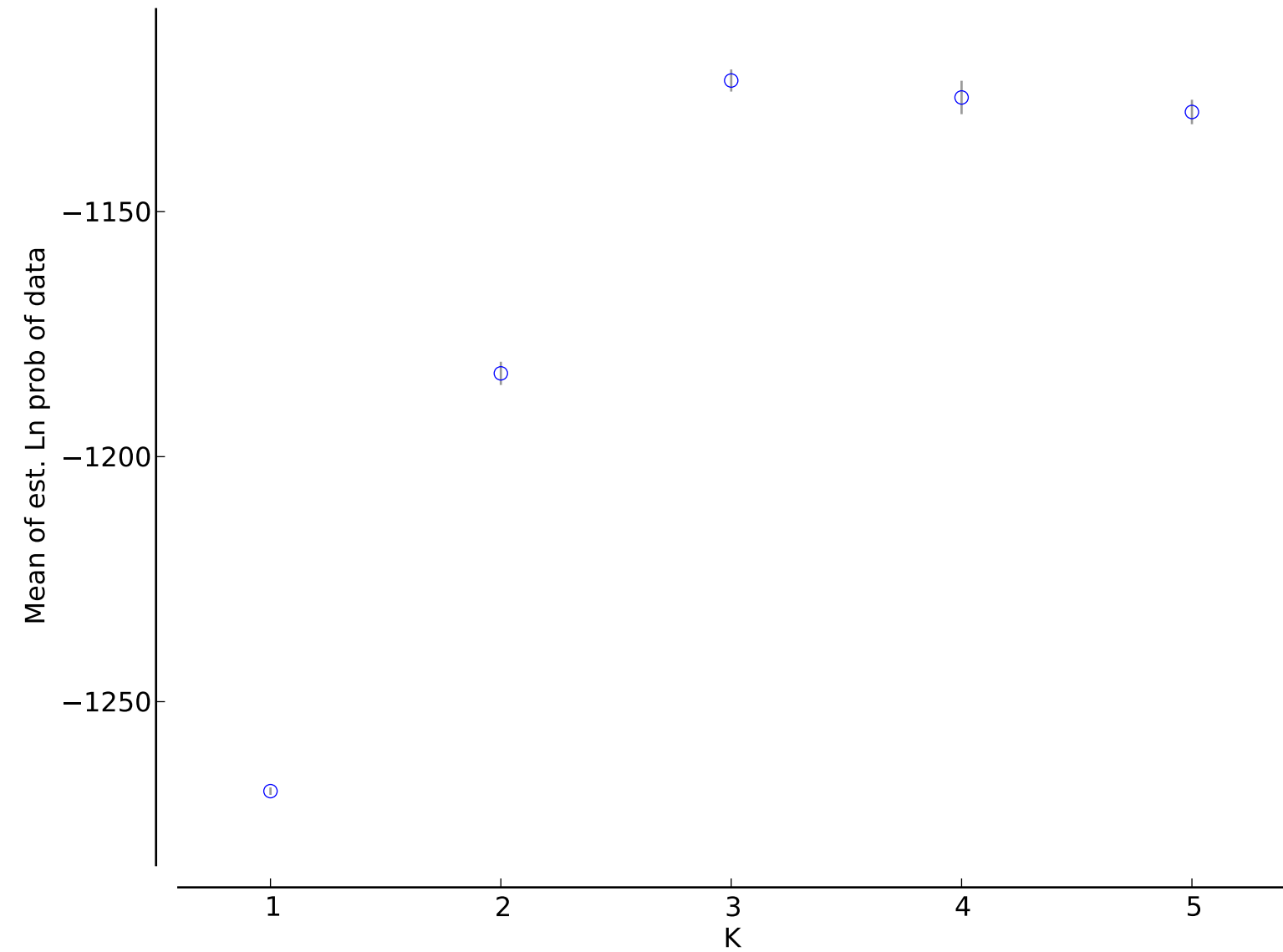

Supplement: S3 Fig — (PDF) [file pone.0186866.s003.pdf]

$$\text{DeltaK} = \text{mean}(|L''(K)|) / \text{sd}(L(K))$$

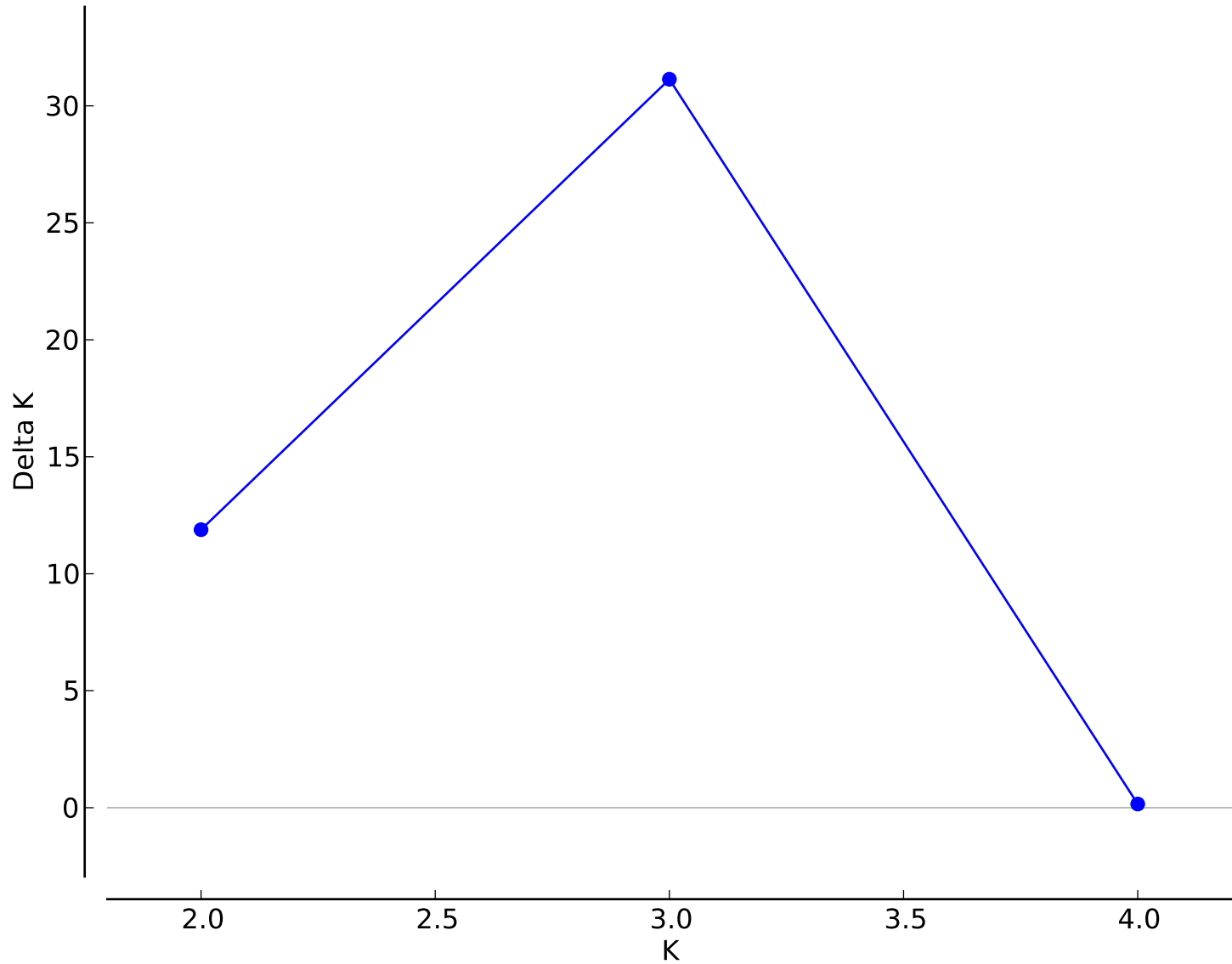

Supplement: S4 Fig — (PDF) [file pone.0186866.s004.pdf]

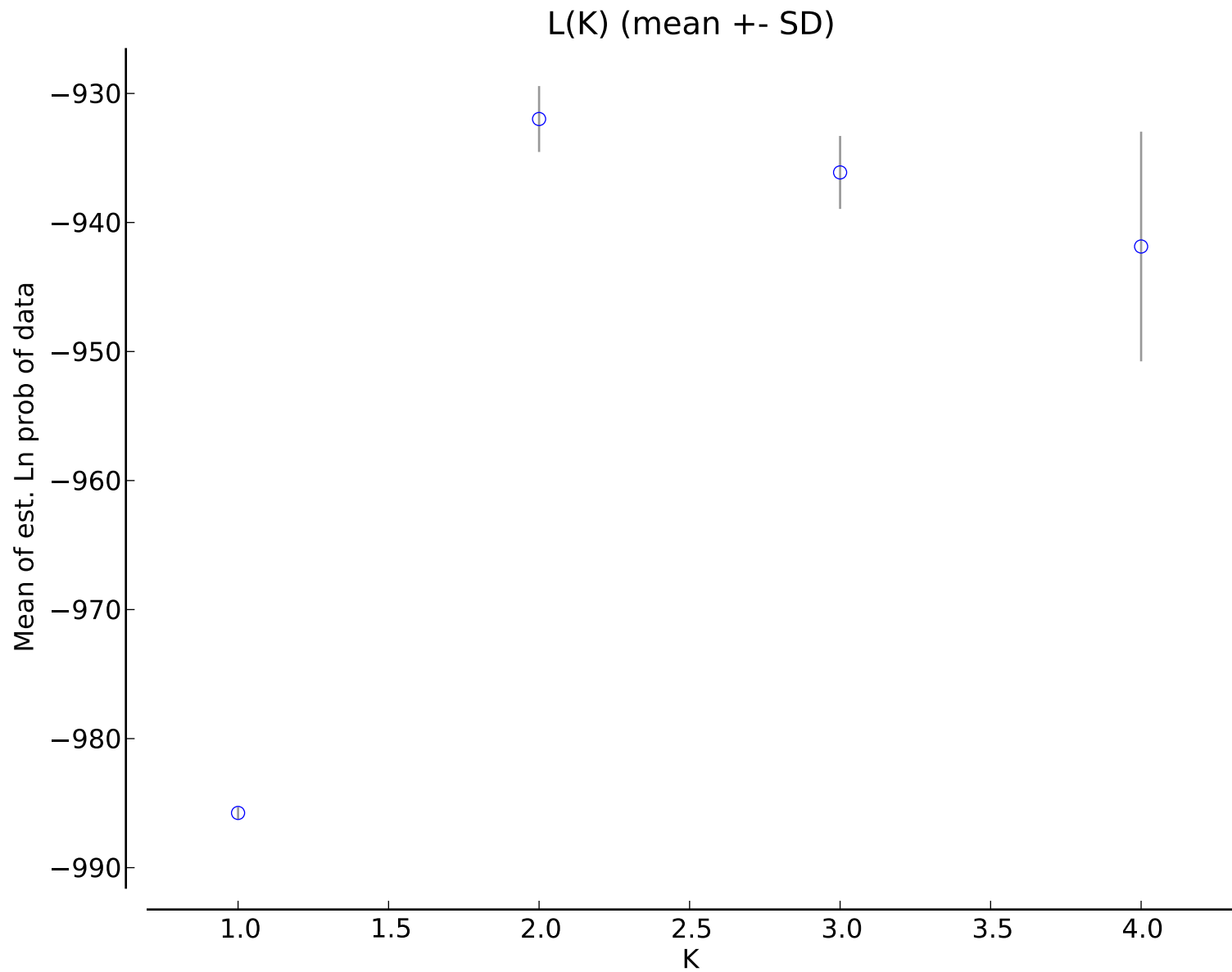

Supplement: S5 Fig — (PDF) [file pone.0186866.s005.pdf]

$$\text{DeltaK} = \text{mean}(|L''(K)|) / \text{sd}(L(K))$$

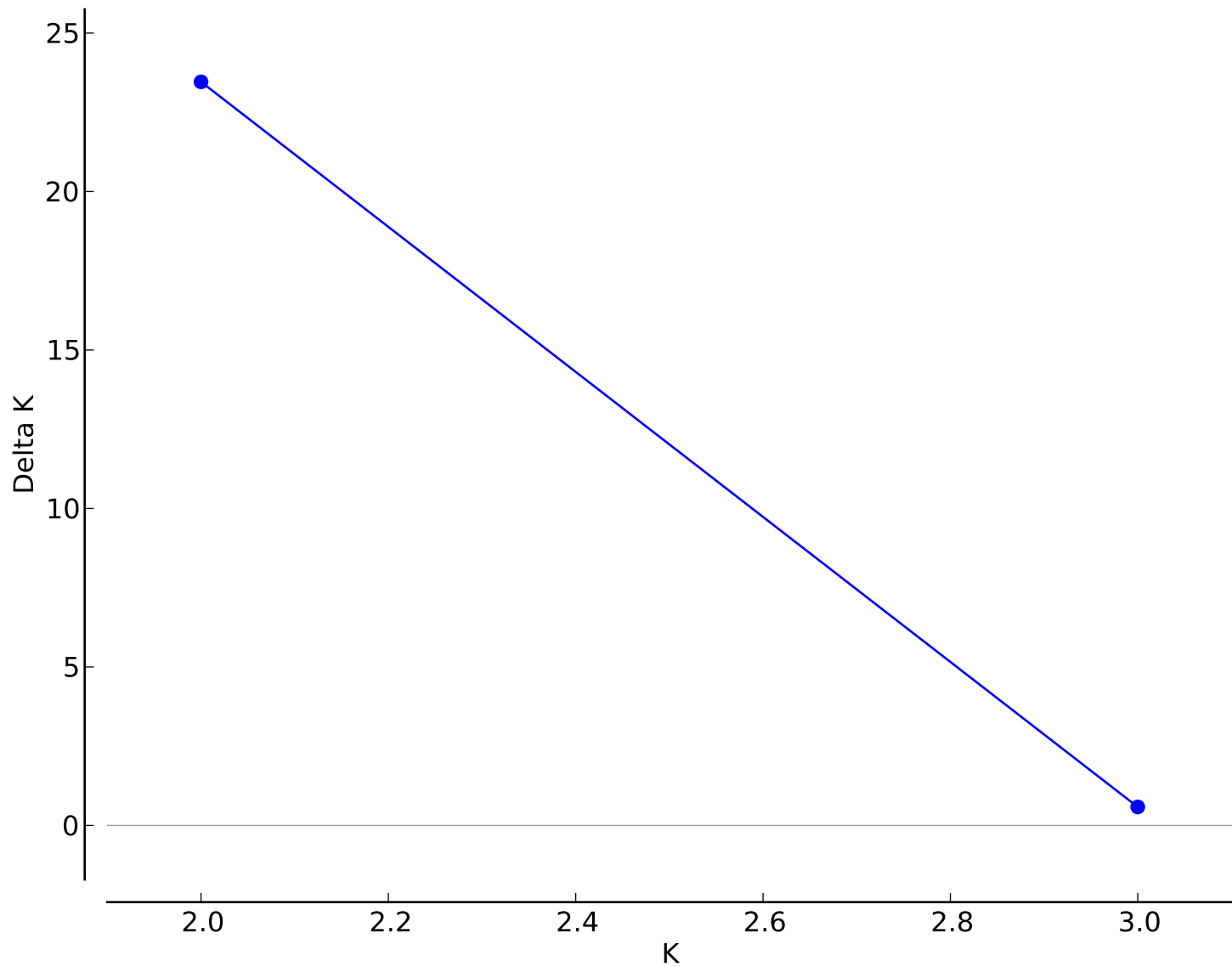

Supplement: S6 Fig — (PDF) [file pone.0186866.s006.pdf]

**Value of BIC  
versus number of clusters**

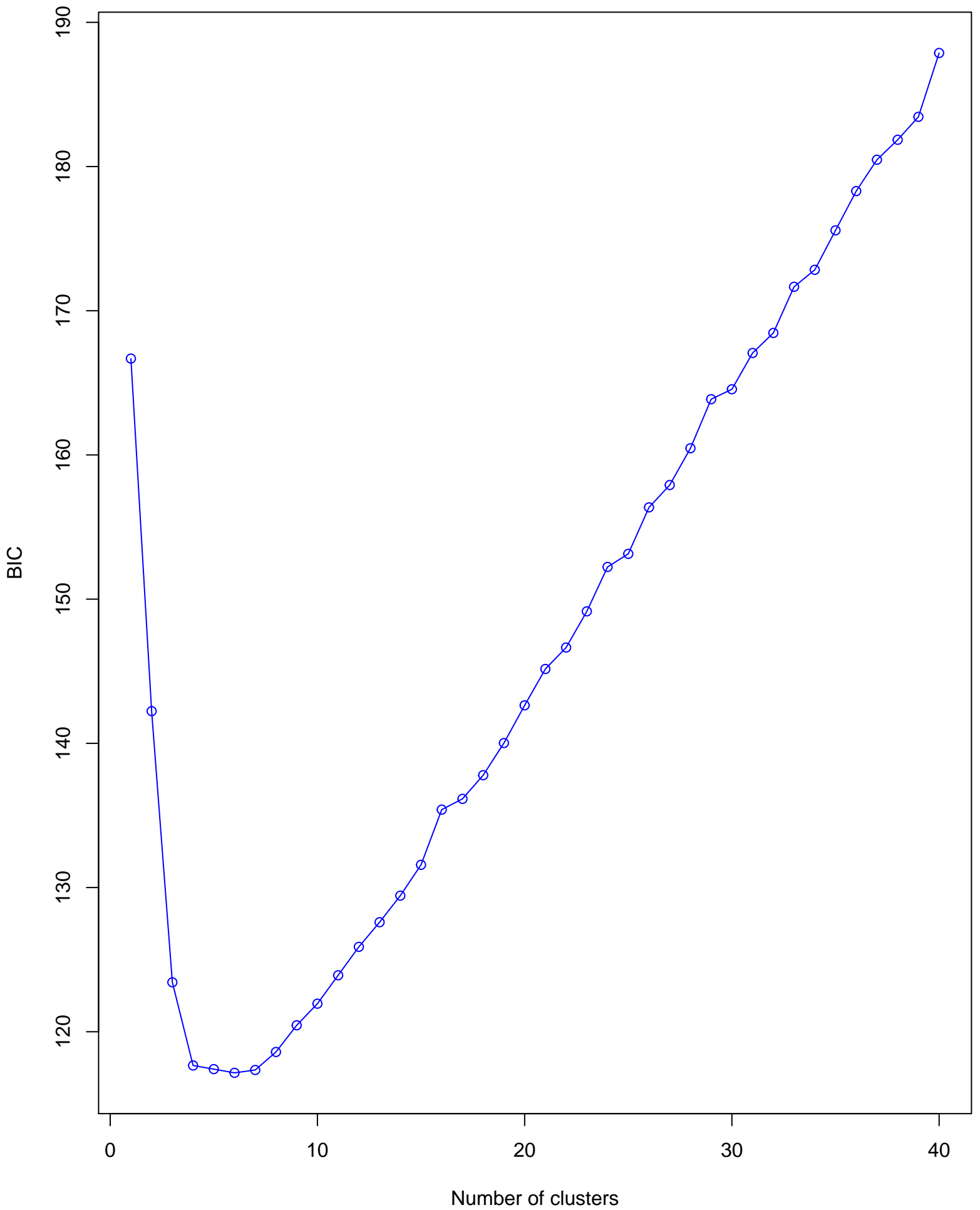

Supplement: S7 Fig — (PDF) [file pone.0186866.s007.pdf]

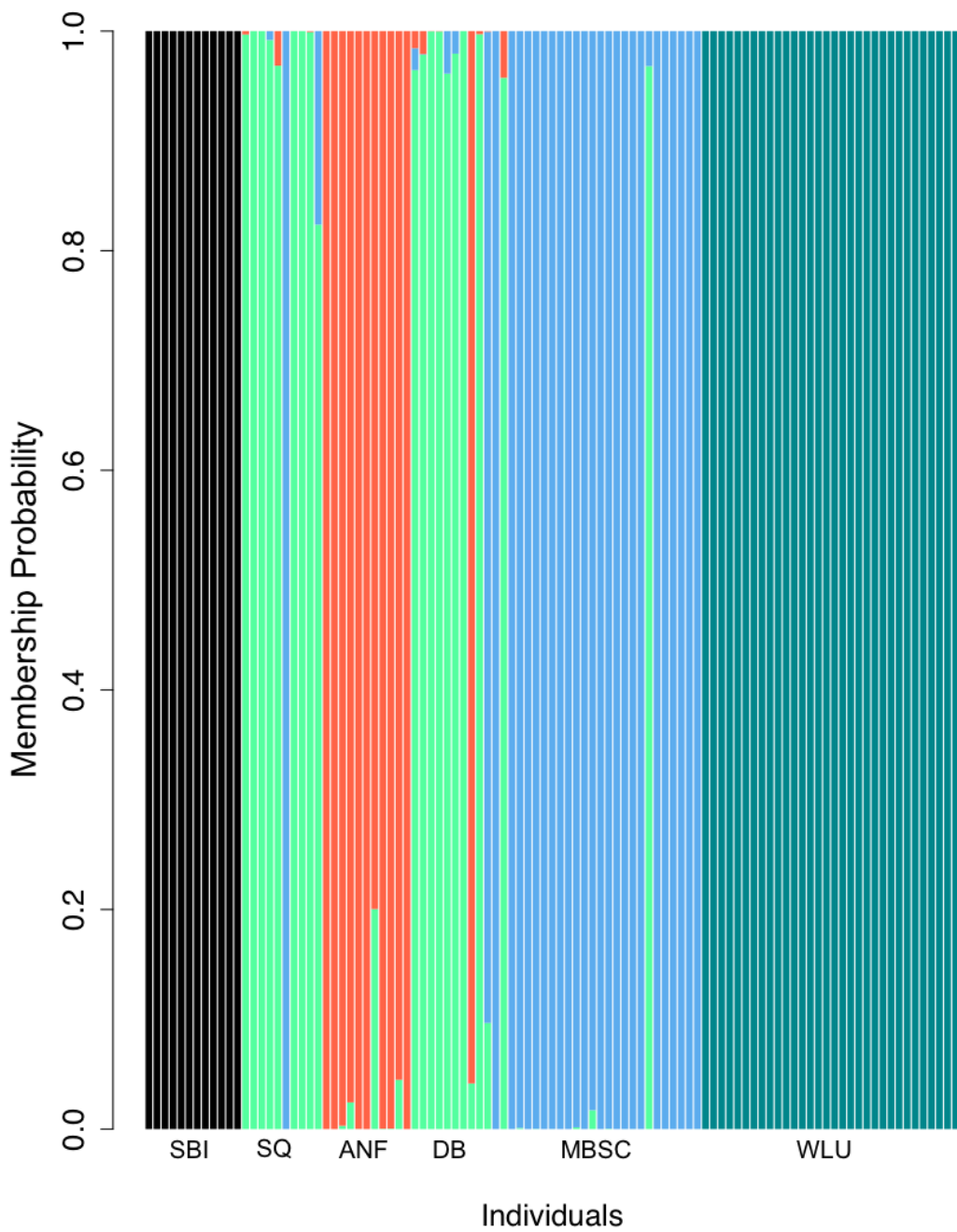

Supplement: S8 Fig — Individuals are represented by a single bar. The number of clusters is K = 5 and colors correspond to those in Fig 5. (PDF) [file pone.0186866.s008.pdf]
