# Supplementary material for: Assessment of intra and interregional genetic variation in the Eastern Red-backed Salamander, Plethodon cinereus, via analysis of novel microsatellite markers
Source: PLoS One. 2017 Oct 20;12(10):e0186866. doi: 10.1371/journal.pone.0186866 (PMC5650168; doi:10.1371/journal.pone.0186866)
Supplement: S1 Table — (DOCX) [file pone.0186866.s015.docx]

| Query (GenBank Accession #) | Locus | Best hit accession ID | Best hit description | Alignment length (bp) | % identity | Bit score | E-value |
| --- | --- | --- | --- | --- | --- | --- | --- |
| MF464607 | PC1 | No hit | N/A | N/A | N/A | N/A | N/A |
| MF464608 | PC2 | No hit | N/A | N/A | N/A | N/A | N/A |
| MF464609 | PC3 | No hit | N/A | N/A | N/A | N/A | N/A |
| MF464610 | PC4 | AC192137^+^ | *Pan troglodytes BAC clone CH251-591O10 from chromosome 4* | 83 | 83 | 86.04 | 8.54 x 10^-13^ |
| MF464611 | PC5 | No hit | N/A | N/A | N/A | N/A | N/A |
| MF464612 | PC6 | AC155327^+^ | *Mus musculus* 6 BAC RP24-176G23 | 120 | 75 | 78.83 | 7.93 x 10^-11^ |
| MF464613 | PC7 | BX936311 ^+^ | Zebrafish DNA sequence from clone DKEY-53B10 in linkage group 22 | 120 | 75 | 84.24 | 1.43 x 10^-12^ |
| MF464614 | PC8 | FP236823^+^ | Zebrafish DNA sequence from clone CH73-189M19 in linkage group 13 | 75 | 86 | 91.45 | 1.50 x 10^-14^ |
| MF464615 | PC9 | HM163335^+^ | *Gehyra variegata* voucher GH236 clone GVN6E microsa-tellite sequence | 64 | 89 | 86.04 | 6.85 x 10^-13^ |
| MF464616 | PC10 | AC121300^+^ | *Mus musculus* chromosome 6, clone RP23-408I12 | 47 | 95 | 77.03 | 4.48 x 10^-10^ |
| MF464617 | PC11 | AC100967^+^ | Mus musculus chromosome 5, clone RP23-74L20 | 171 | 74 | 104.08 | 2.95 x 10^-18^ |
| MF464618 | PC12 | No hit | N/A | N/A | N/A | N/A | N/A |
| MF464619 | PC13 | No hit | N/A | N/A | N/A | N/A | N/A |
| MF464620 | PC14 | No hit | N/A | N/A | N/A | N/A | N/A |
| MF464621 | PC15 | XR_002042199^+^ | PREDICTED: *Larimichthys crocea* nectin-4-like (LOC104935604), transcript variant X8 | 86 | 79 | 69.81 | 7.59 x 10^-8^ |
| MF464622 | PC16 | LL907484^+^ | *Schistocephalus solidus* genome assembly S_solidus_NST_G2 ,scaffold SS LN_scaffold0007103 | 75 | 85 | 86.04 | 1.09 x 10^-12^ |
| MF464623 | PC17 | XM_642530^+^ | *Dictyostelium discoideum* AX4 hypothetical protei-n (DDB_G0268186) mRNA | 181 | 75 | 120.31 | 3.33 x 10^-23^ |
| MF464624 | PC18 | No hit | N/A | N/A | N/A | N/A | N/A |
| MF464625 | PC19 | No hit | N/A | N/A | N/A | N/A | N/A |
| MF464626 | PC20 | JX946138 | *Plethodon albagula* microsatellite PLAL_331b sequ-ence | 148 | 72 | 84.24 | 3.41 x 10^-12^ |
| MF464627 | PC21 | No hit | N/A | N/A | N/A | N/A | N/A |
| MF464628 | PC22 | No hit | N/A | N/A | N/A | N/A | N/A |
| MF464629 | PC23 | BX897687^+^ | Zebrafish DNA sequence from clone DKEY-151O8 | 115 | 74 | 75.22 | 1.78 x 10^-9^ |
| MF464630 | PC24 | LN590706 ^+^ | *Cyprinus carpio* genome assembly common carp genome, scaffold: LG36, chromosome: 36 | 122 | 86 | 138.34 | 1.70 x 10^-28^ |
| MF464631 | PC25 | AC118676 ^+^ | *Mus musculus* chromosome 7, clone RP24-178N18 | 109 | 83 | 109.49 | 5.56 x 10^-20^ |
| MF464632 | PC26 | No hit | N/A | N/A | N/A | N/A | N/A |
| MF464633 | PC27 | LK946504 ^+^ | *Angiostrongylus cantonensis* genome assembly A_cantonensis_China ,scaffold ACAC_scaffold0000684 | 86 | 81 | 80.63 | 3.62 x 10^-11^ |
| MF464634 | PC28 | CU607079 ^+^ | Zebrafish DNA sequence from clone CH73-207H17 in linkage group 12 | 121 | 79 | 104.08 | 2.93 x 10^-18^ |
| MF464635 | PC29 | AY532599 ^+^ | *Plethodon elongatus* microsatellite Plel105 sequence | 123 | 87 | 145.56 | 8.27 x 10^-31^ |
| MF464636 | PC30 | No hit | N/A | N/A | N/A | N/A | N/A |
| MF464637 | PC31 | AL731724^+^ | Mouse DNA sequence from clone RP23-1F19 on chromosome 2 | 51 | 96 | 84.24 | 3.00 x 10^-12^ |
| MF464638 | PC32 | No hit | N/A | N/A | N/A | N/A | N/A |
| MF464639 | PC33 | AY814452^+^ | *Schistosoma japonicum* clone SJCHGC00171 unknown mRNA | 70 | 82 | 69.81 | 8.28 x 10^-8^ |
| MF464640 | PC34 | LL880420^+^ | Schistosoma margrebowiei genome assembly S_margrebowiei_Zambia ,scaffold SMRZ_scaffold0002076 | 130 | 80 | 95.06 | 1.75 x 10^-15^ |
| MF464641 | PC35 | CR846083^+^ | Zebrafish DNA sequence from clone DKEYP-34A6 in linkage group 14 | 56 | 87 | 69.81 | 5.17 x 10^-8^ |
| MF464642 | PC36 | CR854835^+^ | Zebrafish DNA sequence from clone DKEY-69C5 in linkage group 16 | 52 | 94 | 77.03 | 5.51 x 10^-10^ |
| MF464643 | PC37 | No hit | N/A | N/A | N/A | N/A | N/A |
| MF464606 | PC38 | No hit | N/A | N/A | N/A | N/A | N/A |
| MF464644 | PC39 | No hit | N/A | N/A | N/A | N/A | N/A |
| MF464645 | PC40 | LN592962^+^ | *Cyprinus carpio* genome assembly common carp genome ,scaffold 00000251 | 41 | 97 | 69.81 | 7.04 x 10^-8^ |

Searches were conducted using NCBI’s default settings for BLASTn and a critical E-value of 10^-7^.

^+^Alignment regions corresponds entirely or primarily to a microsatellite region.

N/A = not applicable
