# Supplementary material for: Assessment of intra and interregional genetic variation in the Eastern Red-backed Salamander, Plethodon cinereus, via analysis of novel microsatellite markers
Source: PLoS One. 2017 Oct 20;12(10):e0186866. doi: 10.1371/journal.pone.0186866 (PMC5650168; doi:10.1371/journal.pone.0186866)
Supplement: S2 Table — Ninety-five percent confidence intervals are given in parentheses. The symbol ∞ indicates that NeESTIMATOR was unable to estimate Ne and/or associated confidence limits. (DOCX) [file pone.0186866.s016.docx]

| **Population** | **Linkage Disequilibrium** | **Heterozygosity**  **Excess** |
| --- | --- | --- |
| SBI | 65 (2.5 - ∞) | ∞ (9.0 - ∞) |
| SQ | ∞ (5.9 - ∞) | ∞ (5.8 - ∞) |
| DB | ∞ (15.1 - ∞) | ∞ (9.7 - ∞) |
| MBSC | 517.8 (49.7- ∞) | ∞ (∞ - ∞) |
| ANF | 158.3 (8.7- ∞) | ∞ (7.7 - ∞) |
| WLU | ∞ (153.4 - ∞) | ∞ (∞ - ∞) |
